# Olanzapine (10 mg vs 5 mg vs 2.5 mg) for the prophylaxis of chemotherapy-induced nausea and vomiting (CINV) — a systematic review and network meta-analysis

**DOI:** 10.1007/s00520-026-10944-z

**Published:** 2026-07-16

**Authors:** Ronald Chow, Daniel Zhang, Gregory W. Chai, Monica Yuen, Angel Lu, Victoria Fortuna, Sumeet Talwar, Gabriel Boldt, Michael Lock, Shing Fung Lee, Lawson Eng, Hirotoshi Iihara, Mary Louise Affronti, Mitsue Saito, Matti Aapro, Paul J. Hesketh, Florian Scotté, Christina H. Ruhlmann, Jennifer Leigh

**Affiliations:** 1 Temerty Faculty of Medicine, University of Toronto, Toronto, ON Canada; 2 Odette Cancer Centre, Sunnybrook Health Sciences Centre, Toronto, ON Canada; 3 Centre for Evidence-Based Medicine, University of Oxford, Oxford, UK; 4 Princess Margaret Cancer Centre, University Health Network, Toronto, ON Canada; 5 Siebel School of Computing and Data Science, University of Illinois Urbana-Champaign, Champaign, IL USA; 6 Schulich School of Medicine & Dentistry, University of Western Ontario, London, ON Canada; 7 Department of Radiation Oncology, National University Cancer Institute, National University Hospital, Singapore, Singapore; 8 Department of Medicine, Yong Loo Lin School of Medicine, National University of Singapore, Singapore, Singapore; 9 Department of Pharmacy, Gifu University Hospital, 1-1 Yanagido, Gifu, Gifu, 501-1194 Japan; 10 Cancer Center, Gifu University Hospital, 1-1 Yanagido, Gifu, Gifu, 501-1194 Japan; 11 Duke University School of Nursing, Duke University, Durham, NC USA; 12 Juntendo University Graduate School of Medicine, Bunkyo-Ku, Tokyo, 113-8421 Japan; 13 Genolier Cancer Centre, Geneva, Switzerland; 14 Lahey Cancer Institute, Lahey Hospital & Medical Centre, Burlington, MA USA; 15 Département Interdisciplinaire D′Organisation Des Parcours Patients, Gustave-Roussy, Université Paris-Saclay, 94800 Villejuif, France; 16 Department of Oncology, Odense University Hospital, Odense, Denmark

**Keywords:** Olanzapine, Chemotherapy-induced nausea and vomiting, CINV prophylaxis

## Abstract

**Introduction:**

Olanzapine is an established antiemetic for the prevention of chemotherapy-induced nausea and vomiting (CINV), although the optimal dose for balancing efficacy and tolerability remains uncertain. We conducted a systematic review and network meta-analysis comparing olanzapine 2.5 mg, 5 mg, and 10 mg for CINV prophylaxis.

**Methods:**

PubMed, Embase, and Cochrane CENTRAL were searched from inception to April 9, 2026 for randomized controlled trials evaluating olanzapine for CINV prophylaxis in adults receiving chemotherapy. A frequentist random-effects network meta-analysis was performed. Primary outcomes included complete response during the acute, delayed, long-delayed, and overall phases. Secondary outcomes included complete control, no nausea, no vomiting, and safety outcomes.

**Results:**

A total of 54 randomized controlled trials involving 10,455 participants were included. Olanzapine-containing regimens consistently improved complete response, complete control, no nausea, and no vomiting outcomes compared with placebo-controlled regimens across all phases of CINV. Olanzapine 10 mg generally demonstrated the numerically largest efficacy estimates. However, direct comparisons between olanzapine 10 mg and 5 mg did not demonstrate statistically significant differences across efficacy outcomes, suggesting preserved efficacy with dose reduction. In highly emetogenic chemotherapy populations, olanzapine 5 mg demonstrated efficacy comparable to olanzapine 10 mg. In moderately emetogenic chemotherapy populations, olanzapine 5 mg consistently improved outcomes compared with placebo-controlled regimens. Dose reduction from olanzapine 10 mg to 5 mg was not clearly associated with lower sedation risk. Evidence for olanzapine 2.5 mg remained very limited but suggested the possibility of preserved efficacy with lower toxicity.

**Conclusions:**

Olanzapine-containing regimens significantly improved prevention of CINV. Olanzapine 5 mg appeared to preserve substantial efficacy relative to olanzapine 10 mg, although dose reduction did not clearly reduce sedation risk. Additional randomized trials are needed to further define the optimal olanzapine dose.

## Introduction

Chemotherapy-induced nausea and vomiting (CINV) remains one of the most distressing and feared adverse effects of cancer systemic therapy despite substantial advances in antiemetic prophylaxis [1]. Poorly controlled CINV can negatively affect nutritional intake, functional status, quality of life, treatment adherence, and healthcare utilization [1].

Olanzapine, an atypical antipsychotic with antagonistic activity at multiple neurotransmitter receptors implicated in emesis pathways, including dopamine, serotonin, histamine, and muscarinic receptors, has emerged as an important component of modern antiemetic prophylaxis [2]. Randomized trials [3] and meta-analyses [4] have demonstrated that olanzapine improves complete response and nausea control when added to standard antiemetic regimens, particularly among patients receiving highly emetogenic chemotherapy (HEC). As a result, international guidelines such as those from ASCO [5] and MASCC/ESMO [6] now recommend olanzapine-containing regimens in selected patients receiving emetogenic chemotherapy.

Olanzapine 10 mg daily has been the most commonly studied and recommended dose for CINV prophylaxis [4]. However, olanzapine-associated adverse effects, particularly somnolence and sedation, may impair tolerability and limit adherence, especially among older adults and patients with advanced cancer or impaired performance status. In response to these concerns, lower-dose strategies using 5 mg and more recently 2.5 mg have increasingly been adopted in clinical practice in an effort to preserve antiemetic efficacy while minimizing toxicity. Emerging evidence suggests that 5 mg olanzapine may provide comparable control of CINV with less sedation than 10 mg, [7] while preliminary studies are beginning to evaluate whether 2.5 mg may also offer clinically meaningful benefit [8].

Despite growing interest in dose-reduced olanzapine regimens, the optimal dose for balancing efficacy and tolerability remains uncertain. Existing pairwise comparisons are limited, and many studies compare only selected doses or use differing antiemetic backbone regimens, making direct interpretation challenging. A network meta-analysis provides an opportunity to integrate both direct and indirect evidence across available dosing strategies and generate comparative estimates among olanzapine 2.5 mg, 5 mg, and 10 mg.

Therefore, the objective of this systematic review and network meta-analysis is to compare the efficacy and safety of different olanzapine doses for the prophylaxis of CINV in adults receiving chemotherapy. Specifically, we aim to determine whether olanzapine dose reduction can maintain efficacy while reducing toxicity.

## Methods

The review was pre-reigstered [9] and reported in accordance with the Preferred Reporting Items for Systematic Reviews and Meta-Analyses (PRISMA) extension statement for network meta-analyses [10].

### Outcomes

The primary outcome was complete response during the acute (0–24 h post-chemotherapy), delayed (24–120 h), long-delayed (> 120 h) or overall phase of CINV, defined as no emesis and no use of rescue antiemetic medication during the prespecified assessment interval. Secondary efficacy outcomes included complete control (CC) defined as complete response and no worse than mild nausea, absence of nausea and absence of vomiting during the acute, delayed, or overall phases. Safety outcomes included somnolence or sedation, dizziness, constipation, any adverse event, CTCAE grade 3–4 adverse events, and treatment discontinuation due to adverse events.

### Search strategy

An informational specialist (GB) conducted a literature search in PubMed, Embase and the Cochrane Central Register of Controlled Trials (CENTRAL) from database inception to April 9, 2026. The search strategy combined controlled vocabulary terms and keywords related to chemotherapy-induced nausea and vomiting, olanzapine, and randomized controlled trials. Search strategies were adapted for each database. The full search strategy is reported in Appendix 1. No language restrictions were applied.

### Eligibility criteria

Articles were selected for inclusion if they reported on adults aged 18 years or older receiving chemotherapy for any malignancy setting in which olanzapine was administered for the prevention of CINV. Studies evaluating olanzapine administered at any doses (2.5 mg, 5 mg, 10 mg) were eligible for inclusion. Olanzapine could be given at any schedule or duration, and either alone or in combination with standard antiemetic prophylaxis. Only randomized controlled trials were included, to restrict the available data to only the highest level of primary research available.

All search results underwent a two-stage screening process. During title and abstract screening, studies were considered potentially eligible if they evaluated olanzapine as a prophylaxis for CINV. During full-text screening, studies were included if they were randomized controlled trials and met all inclusion criteria.

Screening was conducted independently and in duplicate. One reviewer (DZ) developed a software workflow incorporating ChatGPT-5 to assist with study screening according to the predefined eligibility criteria. For title and abstract screening, the model was provided the abstract, while for full-text screening the complete article text was used. The model was prompted to extract study characteristics and determine eligibility based on the predefined inclusion and exclusion criteria. Outputs included structured standardized machine-readable (JSON-formatted) data extraction, a reasoning summary, and a confidence rating (low, medium, or high). Similar large language model (LLM)-assisted systematic review methodologies have previously demonstrated high sensitivity and specificity comparable to human reviewers [11].

Independent human screening was performed in parallel by a second reviewer (RC). Discordant decisions between the LLM-assisted workflow and human review were examined jointly. The human reviewer assessed the model-generated reasoning, and additional prompting of ChatGPT-5 was performed when clarification of the decision-making process was required. Disagreements were resolved through discussion between human and LLM to achieve consensus. When consensus could not be reached, a third reviewer (JL) adjudicated the decision.

### Data extraction

One human (RC) and LLM (DZ) independently extracted data using standardized data collection forms. Extracted variables included study characteristics, country and setting, publication year, patient population, cancer type, treatment intent, chemotherapy emetogenicity, olanzapine dose and schedule, antiemetic backbone regimen, sample size, outcome definitions, follow-up duration, and reported efficacy and safety outcomes.

### Quality assessment

Studies were evaluated for quality using the Cochrane Risk of Bias 2 (RoB 2) tool [12]. Quality assessment was visually presented using robvis [13].

### Statistical analysis

The geometry of the treatment network was examined graphically. The transitivity assumption was evaluated by assessing the distribution of potential effect modifiers across treatment comparisons, including chemotherapy emetogenicity, cancer type, treatment intent, use of NK1 receptor antagonists, use of 5-HT3 receptor antagonists, dexamethasone schedule, olanzapine duration and timing, and baseline risk of sedation.

A frequentist random-effects network meta-analysis was conducted given the treatment network was connected and assumptions of transitivity were considered clinically reasonable. Treatment effects were summarized using pooled (from both direct and indirect comparisons) odds ratios (ORs) with corresponding 95% confidence intervals (CIs).

Prespecified subgroup analyses was conducted by chemotherapy emetogenicity (HEC versus moderately emetogenic chemotherapy (MEC)). The predefined intervention nodes for the network meta-analysis were olanzapine 2.5 mg, olanzapine 5 mg, olanzapine 10 mg, and placebo or usual care without olanzapine. All analyses were conducted using StataBE 18.1. Type I error was set at 0.05.

## Results

**Fig. 1:**
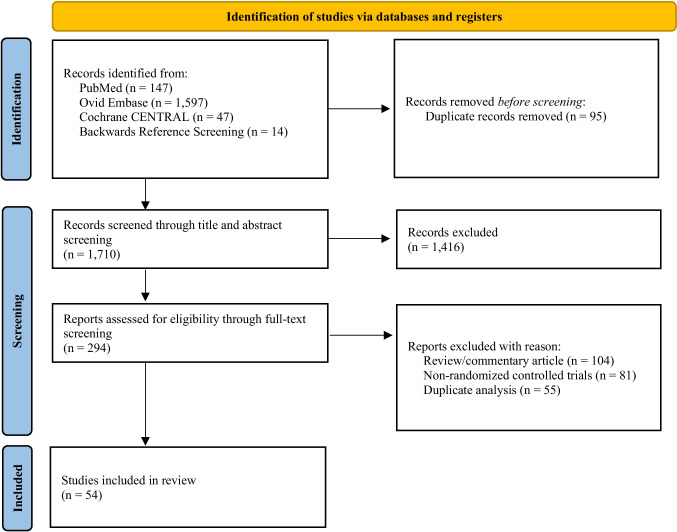
PRISMA flow diagram

**Fig. 2:**
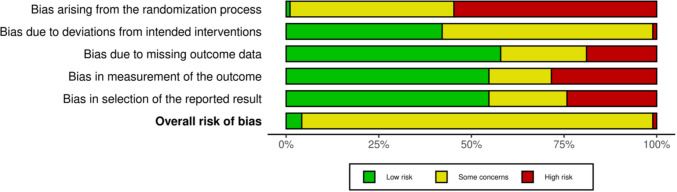
Quality assessment

A total of 1,805 records were identified through database searching and additional sources. After duplicate removal and screening, 54 randomized controlled trials [3, 15–72] involving 11,002 participants were included in the review (Fig. 1). Females comprised 54.7% of the pooled study population. Most studies evaluated highly emetogenic chemotherapy regimens (34 studies), followed by moderately emetogenic chemotherapy (9 studies), while 11 studies enrolled mixed-emetogenicity populations. Across included studies, olanzapine 5 mg was evaluated in 32 studies, olanzapine 10 mg in 26 studies, and olanzapine 2.5 mg in 1 study, noting that some trials evaluated more than one olanzapine dose (Appendix 2). The majority of included trials were judged to have low risk of bias or some concerns (Fig. 2). Studies categorized as having some concerns most commonly lacked sufficient detail regarding allocation concealment, blinding procedures, or outcome assessment methods rather than demonstrating clear methodological flaws.

### Complete response

**Fig. 3:**
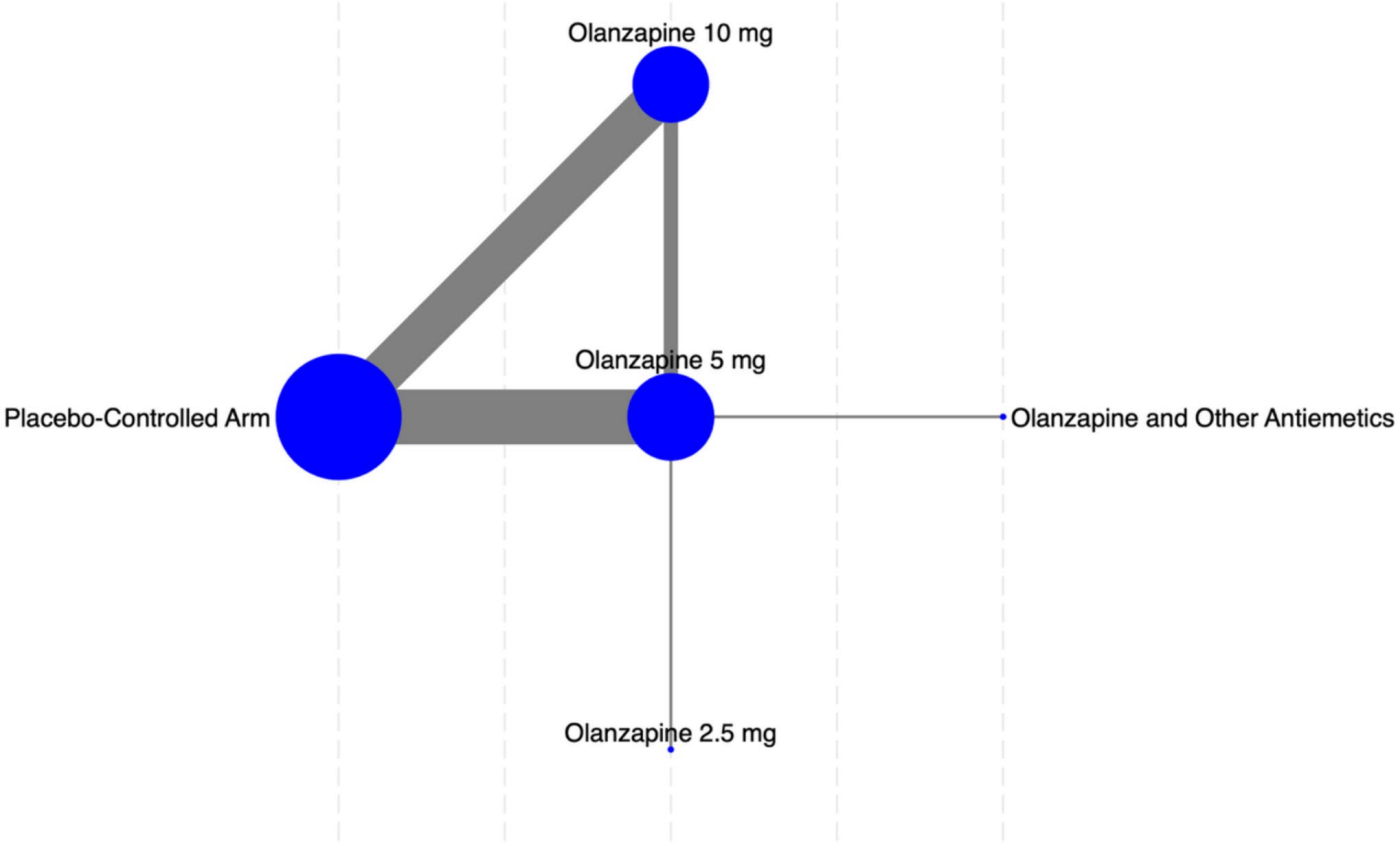
Network map for complete response

Network geometry for complete response is shown in Fig. 3, and pooled network estimates are presented in Table 1. HEC subgroup analyses are presented in Appendix 3, while MEC subgroup analyses are presented in Appendix 4.

**Table 1:** Complete response (odds ratio and 95% confidence intervals). Odds ratios > 1 favor the row-defining treatment

1.1 Acute phase					
	Placebo	OLN10	OLN5	OLN2.5	Other
Placebo	—	0.50 (0.26–0.99)	0.55 (0.28–1.07)	0.53 (0.27–1.03)	0.64 (0.32–1.25)
OLN10	1.98 (1.01–3.88)	—	1.09 (0.55–2.13)	1.04 (0.53–2.04)	1.26 (0.64–2.47)
OLN5	1.82 (0.93–3.57)	0.92 (0.47–1.80)	—	0.96 (0.49–1.88)	1.16 (0.59–2.27)
OLN2.5	1.90 (0.97–3.73)	0.96 (0.49–1.88)	1.04 (0.53–2.05)	—	1.21 (0.62–2.37)
Other	1.57 (0.80–3.08)	0.79 (0.40–1.55)	0.86 (0.44–1.69)	0.83 (0.42–1.62)	—
1.2 Delayed phase					
	Placebo	OLN10	OLN5	OLN2.5	Other
Placebo	—	0.39 (0.20–0.76)	0.46 (0.24–0.89)	0.41 (0.21–0.81)	0.58 (0.30–1.12)
OLN10	2.56 (1.32–5.00)	—	1.18 (0.62–2.23)	1.06 (0.56–2.01)	1.47 (0.77–2.81)
OLN5	2.17 (1.12–4.17)	0.85 (0.45–1.61)	—	0.90 (0.47–1.72)	1.25 (0.66–2.39)
OLN2.5	2.44 (1.24–4.76)	0.94 (0.50–1.80)	1.11 (0.58–2.14)	—	1.39 (0.73–2.67)
Other	1.72 (0.89–3.33)	0.68 (0.36–1.30)	0.80 (0.42–1.52)	0.72 (0.37–1.37)	—
1.3 Long-delayed					
	Placebo	OLN5			
Placebo	—	0.43 (0.29–0.63)			
OLN5	2.35 (1.59–3.45)	—			
1.4 Overall phase					
	Placebo	OLN10	OLN5	OLN2.5	Other
Placebo	—	0.49 (0.33–0.74)	0.72 (0.52–1.00)	0.58 (0.38–0.88)	0.63 (0.43–0.93)
OLN10	2.04 (1.35–3.03)	—	1.46 (1.00–2.13)	1.19 (0.79–1.80)	1.30 (0.87–1.95)
OLN5	1.39 (1.00–1.92)	0.68 (0.47–1.00)	—	0.82 (0.56–1.21)	0.89 (0.61–1.31)
OLN2.5	1.72 (1.14–2.63)	0.84 (0.56–1.27)	1.22 (0.83–1.79)	—	1.09 (0.72–1.64)
Other	1.59 (1.08–2.33)	0.77 (0.51–1.15)	1.12 (0.76–1.65)	0.92 (0.61–1.39)	—

In the acute phase, olanzapine 10 mg significantly improved complete response compared to placebo-controlled regimens (OR 1.98, 95% CI 1.01–3.88). Olanzapine 5 mg (OR 1.82, 95% CI 0.93–3.57) and olanzapine 2.5 mg (OR 1.90, 95% CI 0.97–3.73) also favored olanzapine, although confidence intervals crossed unity. There were no significant differences between olanzapine doses, including comparisons between olanzapine 10 mg and 5 mg (OR 1.09, 95% CI 0.55–2.13) and between olanzapine 10 mg and 2.5 mg (OR 1.04, 95% CI 0.53–2.04). In subgroup analyses, findings were generally consistent in both HEC and MEC populations. In the HEC subgroup, olanzapine 10 mg (OR 2.17, 95% CI 1.10–4.35) and olanzapine 5 mg (OR 2.00, 95% CI 1.02–4.00) significantly improved complete response compared with placebo, whereas, the confidence interval for olanzapine 2.5 mg (OR 1.43, 95% CI 0.55–3.70) crossed unity. In the MEC subgroup, olanzapine 5 mg significantly improved complete response compared with placebo (OR 1.47, 95% CI 1.02–2.17) and olanzapine 10 mg (OR 1.72, 95% CI 0.71–4.17) also favored olanzapine, although confidence interval crossed unity. There were no available data for olanzapine 2.5 mg.in this setting.

In the delayed phase compared with placebo-controlled regimens, olanzapine 10 mg tended to show the largest improvement in complete response (OR 2.56, 95% CI 1.32–5.00), followed by olanzapine 2.5 mg (OR 2.44, 95% CI 1.24–4.76) and olanzapine 5 mg (OR 2.17, 95% CI 1.12–4.17). However, there were again no statistically significant differences between olanzapine doses. Comparisons between olanzapine 10 mg and 5 mg (OR 1.18, 95% CI 0.62–2.23) and between olanzapine 10 mg and 2.5 mg (OR 1.06, 95% CI 0.56–2.01) did not show superiority of one dose over another. Findings remained consistent in subgroup analyses. In the HEC subgroup, both olanzapine 10 mg (OR 2.56, 95% CI 1.28–5.26) and olanzapine 5 mg (OR 2.33, 95% CI 1.19–4.55) significantly improved delayed complete response compared with placebo, whereas, olanzapine 2.5 mg (OR 1.41, 95% CI (0.67–2.94) did not improve delayed complete response significantly. In the MEC subgroup, olanzapine 5 mg remained associated with improved complete response (OR 1.47, 95% CI 1.08–2.00). There were no available data for olanzapine 2.5 mg.in this setting.

In the long-delayed phase, only four studies contributed to the network, and only placebo-controlled and olanzapine 5 mg regimens remained connected. Olanzapine 5 mg significantly improved complete response compared with placebo (OR 2.35, 95% CI 1.59–3.45). Similar findings were observed in both HEC and MEC subgroup analyses.

Across the overall phase compared with placebo-controlled regimens, olanzapine 10 mg demonstrated the strongest effect estimate (OR 2.04, 95% CI 1.35–3.03), followed by olanzapine 2.5 mg (OR 1.72, 95% CI 1.14–2.63), olanzapine-containing regimens combined with other antiemetics (OR 1.59, 95% CI 1.08–2.33), and olanzapine 5 mg (OR 1.39, 95% CI 1.00–1.92). Direct comparisons between olanzapine doses again did not demonstrate statistically significant differences, including comparisons between olanzapine 10 mg and 5 mg (OR 1.46, 95% CI 1.00–2.13) and between olanzapine 10 mg and 2.5 mg (OR 1.19, 95% CI 0.79–1.80). In subgroup analyses, findings were generally similar in HEC and MEC populations. Within the HEC subgroup, olanzapine 10 mg (OR 2.22, 95% CI 1.41–3.45) and olanzapine 5 mg (OR 2.00, 95% CI 1.30–3.13) significantly improved complete response compared with placebo. Olanzapine 2.5 mg (OR 1.41, 95% CI 0.62–3.23) did not demonstrate significant improvement. In the MEC subgroup, olanzapine 5 mg significantly improved complete response compared with placebo (OR 1.47, 95% CI 1.08–2.00), while olanzapine 10 mg showed a numerically favorable but nonsignificant estimate (OR 1.72, 95% CI 0.84–3.57).

### Complete control

Complete control results are presented in Appendix 5. HEC subgroup analyses are presented in Appendix 6, while MEC subgroup analyses are presented in Appendix 7.

In the acute phase, compared with placebo-controlled regimens, olanzapine 10 mg significantly improved complete control (OR 2.04, 95% CI 1.14–3.45). Olanzapine 5 mg also significantly improved complete control (OR 1.41, 95% CI 1.03–1.92), while olanzapine 2.5 mg numerically favored placebo-controlled regimens but did not reach statistical significance (OR 1.49, 95% CI 0.87–2.56). There were no statistically significant differences between olanzapine doses, including comparisons between olanzapine 10 mg and 5 mg (OR 1.45, 95% CI 0.83–2.52) and between olanzapine 10 mg and 2.5 mg (OR 1.36, 95% CI 0.69–2.67). In subgroup analyses, findings were generally similar in both HEC and MEC populations. In the HEC subgroup, olanzapine 10 mg (OR 2.27, 95% CI 1.23–4.17) and olanzapine 5 mg (OR 1.92, 95% CI 1.14–3.23) significantly improved complete control compared with placebo. In the MEC subgroup, olanzapine 5 mg significantly improved complete control compared with placebo (OR 1.47, 95% CI 1.05–2.08).

In the delayed phase, compared with placebo-controlled regimens, olanzapine 10 mg demonstrated the strongest improvement in complete control (OR 2.50, 95% CI 1.47–4.17), followed by olanzapine 2.5 mg (OR 1.69, 95% CI 1.05–2.70) and olanzapine 5 mg (OR 1.52, 95% CI 1.10–2.08). However, there were no statistically significant differences between olanzapine doses. Comparisons between olanzapine 10 mg and 5 mg (OR 1.65, 95% CI 0.96–2.83) and between olanzapine 10 mg and 2.5 mg (OR 1.47, 95% CI 0.78–2.77) did not demonstrate superiority of one dose over another. Findings remained consistent in subgroup analyses. Within the HEC subgroup, olanzapine 10 mg (OR 2.56, 95% CI 1.45–4.55) and olanzapine 5 mg (OR 2.08, 95% CI 1.27–3.45) significantly improved complete control compared with placebo. In the MEC subgroup, olanzapine 5 mg remained associated with improved complete control compared with placebo (OR 1.47, 95% CI 1.08–2.04).

In the long-delayed phase, only placebo-controlled and olanzapine 5 mg regimens remained connected. Olanzapine 5 mg significantly improved complete control compared with placebo (OR 2.78, 95% CI 1.72–4.35). Similar findings were observed in both HEC and MEC subgroup analyses.

Across the overall phase, compared with placebo-controlled regimens, olanzapine 10 mg demonstrated the strongest effect estimate (OR 2.04, 95% CI 1.30–3.13), followed by olanzapine 2.5 mg (OR 1.64, 95% CI 1.06–2.50), olanzapine-containing regimens combined with other antiemetics (OR 1.49, 95% CI 1.00–2.22), and olanzapine 5 mg (OR 1.39, 95% CI 1.02–1.89). Direct comparisons between olanzapine doses again did not demonstrate statistically significant differences, including comparisons between olanzapine 10 mg and 5 mg (OR 1.47, 95% CI 1.00–2.16) and between olanzapine 10 mg and 2.5 mg (OR 1.25, 95% CI 0.82–1.90). In subgroup analyses, findings remained broadly consistent in HEC and MEC populations. Within the HEC subgroup, olanzapine 10 mg (OR 2.33, 95% CI 1.45–3.70) and olanzapine 5 mg (OR 2.00, 95% CI 1.27–3.13) significantly improved complete control compared with placebo. In the MEC subgroup, olanzapine 5 mg significantly improved complete control compared with placebo (OR 1.47, 95% CI 1.08–2.00), while olanzapine 10 mg demonstrated a numerically favorable but nonsignificant estimate (OR 1.72, 95% CI 0.84–3.57).

### No nausea

Nausea results are presented in Appendix 8. HEC subgroup analyses are presented in Appendix 9, while MEC subgroup analyses are presented in Appendix 10.

In the acute phase, compared with placebo-controlled regimens, olanzapine 5 mg significantly improved no nausea outcomes (OR 1.41, 95% CI 1.01–1.96), while olanzapine 10 mg demonstrated a numerically favorable but nonsignificant estimate (OR 2.04, 95% CI 0.98–4.17). Olanzapine 2.5 mg also numerically favored placebo-controlled regimens but did not reach statistical significance (OR 1.45, 95% CI 0.82–2.56). There were no statistically significant differences between olanzapine doses, including comparisons between olanzapine 10 mg and 5 mg (OR 1.45, 95% CI 0.70–3.01) and between olanzapine 10 mg and 2.5 mg (OR 1.40, 95% CI 0.60–3.28). In subgroup analyses, findings were generally similar in both HEC and MEC populations. In the HEC subgroup, olanzapine 10 mg (OR 2.27, 95% CI 1.14–4.55) and olanzapine 5 mg (OR 1.92, 95% CI 1.14–3.33) significantly improved no nausea outcomes compared with placebo. In the MEC subgroup, olanzapine 5 mg significantly improved no nausea outcomes compared with placebo (OR 1.47, 95% CI 1.05–2.08).

In the delayed phase, compared with placebo-controlled regimens, olanzapine 10 mg demonstrated the strongest improvement in no nausea outcomes (OR 2.50, 95% CI 1.49–4.17), followed by olanzapine 5 mg (OR 1.96, 95% CI 1.41–2.70) and olanzapine 2.5 mg (OR 1.79, 95% CI 1.05–3.03). However, there were no statistically significant differences between olanzapine doses. Comparisons between olanzapine 10 mg and 5 mg (OR 1.29, 95% CI 0.74–2.26) and between olanzapine 10 mg and 2.5 mg (OR 1.40, 95% CI 0.70–2.81) did not demonstrate superiority of one dose over another. Findings remained consistent in subgroup analyses. Within the HEC subgroup, olanzapine 10 mg (OR 2.56, 95% CI 1.35–5.00) and olanzapine 5 mg (OR 2.33, 95% CI 1.35–4.00) significantly improved no nausea outcomes compared with placebo. In the MEC subgroup, olanzapine 5 mg remained associated with improved no nausea outcomes compared with placebo (OR 1.47, 95% CI 1.08–2.04).

In the long-delayed phase, only placebo-controlled and olanzapine 5 mg regimens remained connected within the network. Olanzapine 5 mg significantly improved no nausea outcomes compared with placebo (OR 2.33, 95% CI 1.54–3.45). Similar findings were observed in both HEC and MEC subgroup analyses.

Across the overall phase, compared with placebo-controlled regimens, olanzapine 10 mg demonstrated the strongest effect estimate (OR 2.50, 95% CI 1.67–3.70), followed by olanzapine 5 mg (OR 1.96, 95% CI 1.45–2.63) and olanzapine 2.5 mg (OR 1.72, 95% CI 1.12–2.63). Direct comparisons between olanzapine doses again did not demonstrate statistically significant differences, including comparisons between olanzapine 10 mg and 5 mg (OR 1.27, 95% CI 0.82–1.96) and between olanzapine 10 mg and 2.5 mg (OR 1.45, 95% CI 0.88–2.39). In subgroup analyses, findings remained broadly consistent in HEC and MEC populations. Within the HEC subgroup, olanzapine 10 mg (OR 2.50, 95% CI 1.54–4.00) and olanzapine 5 mg (OR 2.22, 95% CI 1.39–3.57) significantly improved no nausea outcomes compared with placebo. In the MEC subgroup, olanzapine 5 mg significantly improved no nausea outcomes compared with placebo (OR 1.47, 95% CI 1.08–2.00), while olanzapine 10 mg demonstrated a numerically favorable but nonsignificant estimate (OR 1.72, 95% CI 0.84–3.57).

### No vomiting

No vomiting results are presented in Appendix 11. HEC subgroup analyses are presented in Appendix 12, while MEC subgroup analyses are presented in Appendix 13.

In the acute phase, compared with placebo-controlled regimens, olanzapine 10 mg significantly improved no vomiting outcomes (OR 2.50, 95% CI 1.49–4.17), while olanzapine 5 mg also demonstrated significant benefit (OR 1.82, 95% CI 1.32–2.50). Olanzapine 2.5 mg numerically favored placebo-controlled regimens but did not reach statistical significance (OR 1.64, 95% CI 0.97–2.78). There were no statistically significant differences between olanzapine doses, including comparisons between olanzapine 10 mg and 5 mg (OR 1.38, 95% CI 0.80–2.39) and between olanzapine 10 mg and 2.5 mg (OR 1.53, 95% CI 0.78–3.00). In subgroup analyses, findings were generally similar in both HEC and MEC populations. In the HEC subgroup, olanzapine 10 mg (OR 2.56, 95% CI 1.35–4.76) and olanzapine 5 mg (OR 2.08, 95% CI 1.27–3.45) significantly improved no vomiting outcomes compared with placebo. In the MEC subgroup, olanzapine 5 mg significantly improved no vomiting outcomes compared with placebo (OR 1.47, 95% CI 1.05–2.08).

In the delayed phase, compared with placebo-controlled regimens, olanzapine 10 mg demonstrated the strongest improvement in no vomiting outcomes (OR 2.50, 95% CI 1.52–4.17), followed by olanzapine 5 mg (OR 1.96, 95% CI 1.45–2.70) and olanzapine 2.5 mg (OR 1.75, 95% CI 1.05–2.94). However, there were no statistically significant differences between olanzapine doses. Comparisons between olanzapine 10 mg and 5 mg (OR 1.28, 95% CI 0.74–2.22) and between olanzapine 10 mg and 2.5 mg (OR 1.43, 95% CI 0.73–2.80) did not demonstrate superiority of one dose over another. Findings remained consistent in subgroup analyses. Within the HEC subgroup, olanzapine 10 mg (OR 2.56, 95% CI 1.41–5.00) and olanzapine 5 mg (OR 2.22, 95% CI 1.35–3.70) significantly improved no vomiting outcomes compared with placebo. In the MEC subgroup, olanzapine 5 mg remained associated with improved no vomiting outcomes compared with placebo (OR 1.47, 95% CI 1.08–2.04).

In the long-delayed phase, only placebo-controlled and olanzapine 5 mg regimens remained connected within the network. Olanzapine 5 mg significantly improved no vomiting outcomes compared with placebo (OR 2.17, 95% CI 1.43–3.33). Similar findings were observed in both HEC and MEC subgroup analyses.

Across the overall phase, compared with placebo-controlled regimens, olanzapine 10 mg demonstrated the strongest effect estimate (OR 2.50, 95% CI 1.69–3.70), followed by olanzapine 5 mg (OR 1.92, 95% CI 1.45–2.56) and olanzapine 2.5 mg (OR 1.69, 95% CI 1.11–2.56). Direct comparisons between olanzapine doses again did not demonstrate statistically significant differences, including comparisons between olanzapine 10 mg and 5 mg (OR 1.29, 95% CI 0.84–1.99) and between olanzapine 10 mg and 2.5 mg (OR 1.47, 95% CI 0.89–2.43). In subgroup analyses, findings remained broadly consistent in HEC and MEC populations. Within the HEC subgroup, olanzapine 10 mg (OR 2.50, 95% CI 1.56–4.17) and olanzapine 5 mg (OR 2.17, 95% CI 1.39–3.45) significantly improved no vomiting outcomes compared with placebo. In the MEC subgroup, olanzapine 5 mg significantly improved no vomiting outcomes compared with placebo (OR 1.47, 95% CI 1.08–2.00), while olanzapine 10 mg demonstrated a numerically favorable but nonsignificant estimate (OR 1.72, 95% CI 0.84–3.57).

### Safety

Safety outcomes are presented in Appendix 14. Compared with placebo-controlled regimens, olanzapine 10 mg was not associated with statistically significant increases in most adverse events, including appetite loss, constipation, diarrhea, fatigue, headache, hiccups, or insomnia. However, olanzapine 10 mg demonstrated a numerically increased risk of sedation compared with placebo-controlled regimens (OR 1.64, 95% CI 0.94–2.86), although this did not reach statistical significance. Olanzapine 10 mg and 5 mg had equivalent risk of sedation (OR 0.69, 95% CI 0.36–1.33). Olanzapine 2.5 mg generally demonstrated numerically lower risks of adverse events than higher-dose olanzapine regimens, although confidence intervals were wide because of limited available data.

## Discussion

In this systematic review and network meta-analysis including 54 randomized controlled trials involving 10,445 patients underwent highly emetogenic chemotherapy or moderately emetogenic chemotherapy, olanzapine-containing antiemetic regimens consistently improved complete response, complete control, no nausea, and no vomiting outcomes compared with placebo-controlled regimens across the acute, delayed, long-delayed, and overall phases of chemotherapy-induced nausea and vomiting. Across efficacy outcomes, olanzapine 10 mg generally demonstrated the numerically largest effect estimates. However, direct comparisons between olanzapine doses did not demonstrate statistically significant differences, suggesting that dose reduction from 10 to 5 mg may preserve antiemetic efficacy.

The most clinically important question addressed by this analysis was whether olanzapine dose reduction can maintain efficacy while reducing toxicity. Among patients receiving HEC, both olanzapine 10 mg and olanzapine 5 mg consistently improved efficacy outcomes compared with placebo-controlled regimens across complete response, complete control, no nausea, and no vomiting endpoints. Importantly, direct comparisons between olanzapine 10 mg and 5 mg did not demonstrate significant differences in efficacy across phases. These findings suggest that olanzapine 5 mg may provide efficacy comparable to olanzapine 10 mg in HEC settings. However, contrary to expectations, dose reduction from 10 to 5 mg was not clearly associated with lower sedation risk. This may be multifactorial, as trials studying 5 mg tended to have a relatively higher proportion of cisplatin-centred regimens and there was non-uniform dexamethasone exposure (ie single day dexamethasone, or modified doses). Taken together, these findings suggest that olanzapine 5 mg may preserve efficacy in HEC populations, although no clear safety advantage has been demonstrated apart from reduced cost-related toxicity.

Within patients receiving MEC, findings differed somewhat from those observed in HEC populations. Across efficacy outcomes, olanzapine 5 mg consistently demonstrated significant benefit compared with placebo-controlled regimens, whereas olanzapine 10 mg frequently demonstrated numerically favorable but nonsignificant estimates. These findings likely reflect smaller sample sizes, the lack of compliance data for antiemetics, the heterogeneity of emetic risk within MEC populations and more limited direct evidence for olanzapine 10 mg in MEC settings, rather than a clear inferiority of the 10 mg dose. Nonetheless, the consistency of benefit observed with olanzapine 5 mg across MEC outcomes supports the clinical use of lower-dose olanzapine in moderately emetogenic chemotherapy populations. Similar to the HEC analyses, however, dose reduction from 10 to 5 mg was not clearly associated with lower rates of sedation or other adverse events.

An additional important finding from this network meta-analysis relates to the emerging role of olanzapine 2.5 mg. Evidence for olanzapine 2.5 mg remained sparse and was informed by one study comprising primarily female patients receiving doxorubicin-cyclophosphamide [14]. These findings suggest that olanzapine 2.5 mg may potentially preserve antiemetic efficacy while improving tolerability, but the certainty of evidence remains limited.

Our findings build upon prior pairwise meta-analyses demonstrating the efficacy of olanzapine for CINV prophylaxis [4]. Previous studies have suggested that olanzapine 5 mg may provide similar efficacy with less sedation compared with olanzapine 10 mg [7]. However, direct head-to-head evidence remains limited. By integrating both direct and indirect evidence across available dosing strategies, the present network meta-analysis provides a more comprehensive comparison of olanzapine dosing approaches and highlights the relative paucity of high-quality comparative data evaluating lower olanzapine doses.

This analysis has several limitations. First, although the overall evidence base was large, direct comparisons between olanzapine doses remained limited, especially for olanzapine 2.5 mg. Second, many safety analyses were characterized by sparse networks and wide confidence intervals, limiting the precision of comparative safety estimates. Finally, several studies were judged to have some concerns for risk of bias because of incomplete reporting of allocation concealment, blinding procedures, or outcome assessment methods.

Future studies should prioritize adequately powered randomized comparisons between olanzapine 10 mg, 5 mg, and 2.5 mg regimens, particularly within homogeneous chemotherapy populations and standardized antiemetic backbones. Additional prospective studies evaluating patient-reported sedation, quality of life, functional outcomes, and older adult populations would also help clarify the optimal balance between efficacy and tolerability. In particular, the preliminary signal suggesting that olanzapine 2.5 mg may preserve efficacy while potentially reducing toxicity warrants further dedicated investigation.

In conclusion, olanzapine-containing regimens significantly improved prevention of chemotherapy-induced nausea and vomiting compared with placebo-controlled regimens across efficacy outcomes. Olanzapine 5 mg appeared to preserve substantial efficacy relative to olanzapine 10 mg in both HEC and MEC settings, although dose reduction did not significantly reduce sedation risk in the current evidence base. Evidence for olanzapine 2.5 mg remains very limited but suggests the possibility of maintained efficacy with improved tolerability. Additional randomized trials are needed to further define the optimal olanzapine dose for balancing antiemetic efficacy and toxicity.

## Data Availability

No datasets were generated or analysed during the current study.

## Appendix 1

### Search strategy

PubMed (147)

(Randomized controlled trials as topic[mh] OR randomized controlled trial[pt] OR randomized[tw] OR randomised[tw])

AND (naus*[tw] OR nausea[mh] OR vomit*[tw] OR vomiting[mh])

AND (olanzapine[mh] OR olanzapine[tw] OR zolafren[tw])

AND (chemotherapy[tw] OR Antineoplastic Agents[mh] OR drug therapy[sh] OR Antineoplastic Combined Chemotherapy Protocols[mh])

EMBASE (1,597) & Cochrane Library (47)

(exp "randomized controlled trial (topic)"/or exp randomized controlled trial/or randomized controlled trial.mp. or randomized.mp. or randomised.mp.)

and (exp nausea/or naus*.mp. or vomit*.mp. or exp vomiting/)

and (olanzapine.mp. or exp olanzapine/or zolafren.mp.)

and (chemotherapy.mp. or exp chemotherapy/or exp antineoplastic agent/or exp drug therapy/or exp antineoplastic agent/)

Remove Medline Records

Limit Cochrane Library

## Appendix 2

See Table 2

**Table 2:** Study characteristics

Study	Sample size	Chemotherapy regimen	Chemotherapy emetogenicity	Antiemetic regimens	
An et al. [15]	117	Carboplatin-containing combination chemotherapy; Carboplatin IV day 1	Moderate	Olanzapine 5 mg PO days 1–4 + Tropisetron 5 mg IV day 1 + Dexamethasone 10 mg IV day 1; OLZ triple regimen	Tropisetron 5 mg IV day 1 + Dexamethasone 10 mg IV day 1; Standard therapy
Attili et al. [16]	82	Cisplatin-based chemotherapy; cisplatin	High	Olanzapine 10 mg oral day before chemotherapy to day 3 post-chemotherapy + granisetron 3 mg IV + dexamethasone 16 mg IV; Group A	Placebo day before chemotherapy to day 3 post-chemotherapy + granisetron 3 mg IV + dexamethasone 16 mg IV; Group B
Babu et al. [17]	100	Mixed highly emetogenic chemotherapy regimens	Mixed	Olanzapine 10 mg PO Day 1, 30–60 min prior to chemotherapy + Olanzapine 5 mg BD PO Days 2–4 + Palonosetron 0.25 mg IV Day 1, 30–60 min prior to chemotherapy + Dexamethasone 20 mg IV Day 1, 30–60 min prior to chemotherapy + Dexamethasone 4 mg BD PO Days 2–4; OPD	Aprepitant 125 mg PO Day 1, 30–60 min prior to chemotherapy + Aprepitant 80 mg OD PO Days 2–3 + Palonosetron 0.25 mg IV Day 1, 30–60 min prior to chemotherapy + Dexamethasone 12 mg IV Day 1, 30–60 min prior to chemotherapy + Dexamethasone 4 mg BD PO Days 2–4; APD
Bajpai et al. [14]	275	Anthracycline-cyclophosphamide or high-dose cisplatin chemotherapy; Anthracycline + Cyclophosphamide + Cisplatin high-dose	High	Olanzapine 10 mg through day 4 + 5-HT3 receptor antagonist + Dexamethasone single dose day 1 only; no delayed doses + NK1 receptor antagonist; Standard 10 mg arm	Olanzapine 2.5 mg through day 4 + 5-HT3 receptor antagonist + Dexamethasone single dose day 1 only; no delayed doses + NK1 receptor antagonist; Experimental 2.5 mg arm
Bhargave et al. [18]	214	Single-day carboplatin-containing chemotherapy, first cycle (most commonly paclitaxel + carboplatin); Carboplatin IV day 1, single-day chemotherapy	Moderate	Olanzapine 5 mg PO once daily days 1–4; started 30 min before chemotherapy on day 1 + Ondansetron 8 mg IV day 1, once daily + Dexamethasone 12 mg IV (20 mg with paclitaxel-containing regimen) IV day 1, once daily; OOD	Fosaprepitant 150 mg IV day 1, once daily + Ondansetron 8 mg IV day 1, once daily + Dexamethasone 12 mg IV (20 mg with paclitaxel-containing regimen) IV day 1, once daily; FOD
Celio et al. [19]	81	Carboplatin/paclitaxel; Paclitaxel 175 mg/m2 IV day 1, 3-h infusion + Carboplatin AUC 5 IV day 1, 60-min infusion, after paclitaxel; q21 days	Moderate	Olanzapine 10 mg PO once daily on days 2–3 + Dexamethasone 4 mg PO once daily on days 2–3; Olanzapine + dexamethasone arm	Dexamethasone 4 mg PO twice daily on days 2–3; Dexamethasone arm
Chen et al. [20]	30	Highly emetogenic chemotherapy (unspecified)	High	Olanzapine 5 mg oral once daily for 4 days, starting the night before chemotherapy + Ondansetron standard dose + Dexamethasone standard dose; Olanzapine triplet	Aprepitant standard dose + Ondansetron standard dose + Dexamethasone standard dose; Aprepitant triplet
Chen et al. [21]	439	Epirubicin plus cyclophosphamide (EC); epirubicin + cyclophosphamide	High	Olanzapine + fosaprepitant + tropisetron + dexamethasone; Quadruple group/Group A control arm	Olanzapine + fosaprepitant + tropisetron; Triple group/Group B experimental arm
Chen et al. [22]	275	Highly emetogenic chemotherapy (predominantly cisplatin-containing regimens); Cisplatin 70 mg/m2 + Doxorubicin 60 mg/m^2^ + Cyclophosphamide 1.5 g/m^2^	High	Olanzapine 5 mg oral once daily at bedtime, days 1–4 + Ondansetron 16 mg IV day 1; F-Group	Olanzapine 5 mg oral once daily at bedtime, days 1–4 + Ondansetron 16 mg IV day 1 + Dexamethasone 10 mg IV days 1–3, administered as a 30-min infusion in 5% glucose 100 ml; D-Group
Clemmons et al. [23]	108	Mixed hematologic malignancy chemotherapy and HCT conditioning regimens	Mixed	Olanzapine 10 mg oral each chemotherapy day plus 3 additional days after chemotherapy + Fosaprepitant 150 mg IV on 1 day + Ondansetron 8–16 mg PO/IV each day of chemotherapy; on TBI days 8 mg PO before each session + Dexamethasone 8–20 mg PO/IV each day of chemotherapy; on TBI days 4 mg PO before each session; FOND-O	Fosaprepitant 150 mg IV on 1 day + Ondansetron 8–16 mg PO/IV each day of chemotherapy; on TBI days 8 mg PO before each session + Dexamethasone 8–20 mg PO/IV each day of chemotherapy; on TBI days 4 mg PO before each session; FOND
Clemons et al. [24]	229	Neo/adjuvant AC, FEC, or TCH	Mixed	Olanzapine 5 mg PO once daily days 1–4 + Aprepitant 125 mg day 1; 80 mg days 2–3 PO once daily day 1, then once daily days 2–3 + Ondansetron 8 mg PO BID on day 1 + Dexamethasone 12 mg before chemotherapy; 4 mg days 2–3 IV then PO 12 mg IV 1 h before chemotherapy, then 4 mg PO BID days 2–3; Experimental	Aprepitant 125 mg day 1; 80 mg days 2–3 PO once daily day 1, then once daily days 2–3 + Ondansetron 8 mg PO BID on day 1 + Dexamethasone 12 mg before chemotherapy; 4 mg days 2–3 IV then PO 12 mg IV 1 h before chemotherapy, then 4 mg PO BID days 2–3 + Placebo PO once daily days 1–4; Control/placebo
Dulal et al. [25]	65	Highly emetogenic chemotherapy: cisplatin-based or anthracycline/cyclophosphamide-based; Cisplatin + Cyclophosphamide + Doxorubicin	High	Olanzapine 10 mg oral 30–60 min before chemotherapy on day 1, then daily on days 2–4 + Ondansetron 16 mg IV day 1, 30–60 min before chemotherapy + Dexamethasone 12 mg IV day 1, 30–60 min before chemotherapy; OLN	Haloperidol 1 mg oral 30–60 min before chemotherapy on day 1 + Haloperidol 0.5 mg twice daily oral days 2–4 + Ondansetron 16 mg IV day 1, 30–60 min before chemotherapy + Dexamethasone 12 mg IV day 1, 30–60 min before chemotherapy; HAL
Gao et al. [26]	132	3-day cisplatin-based doublet regimens; cisplatin 25 mg/m^2^/day IV days 1–3 + gemcitabine + docetaxel + etoposide + pemetrexed + paclitaxel + capecitabine + irinotecan + bevacizumab sometimes added + trastuzumab sometimes added	High	Olanzapine 5 mg PO days 1–3 + aprepitant 125 mg day 1; 80 mg days 2–3 PO day 1, then days 2–3 + tropisetron 5 mg IV days 1–3 + dexamethasone 5 mg PO/IV (reported inconsistently) days 1–3; Quadruple group	Olanzapine 5 mg PO days 1–3 + tropisetron 5 mg IV days 1–3 + dexamethasone 10 mg PO/IV (reported inconsistently) days 1–3; Triplet group
Gedela et al. [27]	122	Mixed highly emetogenic chemotherapy regimens; Anthracycline IV + Cyclophosphamide IV + Paclitaxel IV + Carboplatin AUC‚ ≥4 IV + Cisplatin IV + Dacarbazine IV	Mixed	Olanzapine 10 mg oral at bedtime, days 1–4 + Palonosetron 0.25 mg IV day 1, 30 min before chemotherapy + Dexamethasone 12 mg IV day 1, 30 min before chemotherapy; OPD	Aprepitant 125 mg on day 1; 80 mg on days 2–3 oral day 1; days 2–3 + Palonosetron 0.25 mg IV day 1, 30 min before chemotherapy + Dexamethasone 12 mg IV day 1, 30 min before chemotherapy; APD
Gowda et al. [28]	49	Anthracycline/cyclophosphamide-based chemotherapy for breast cancer (EC, AC, FEC); Doxorubicin 60 mg/m^2^ IV day 1 (AC regimen) + Epirubicin 100 mg/m^2^ IV day 1 (EC/FEC regimen) + Cyclophosphamide 500–600 mg/m^2^ IV day 1 + 5-Fluorouracil 600 mg/m^2^ IV day 1 (FEC regimen)	High	Olanzapine 5 mg oral once daily, days 1–4 + Thalidomide 50 mg oral once daily, days 1–5; first dose with premedications before chemotherapy + Aprepitant 125 mg on day 1, then 80 mg on days 2–3 oral once daily + Ondansetron 8 mg IV then oral 8 mg IV 30 min before chemotherapy on day 1, then 8 mg orally three times daily on days 2–4 + Dexamethasone 8 mg on day 1, then 4 mg on days 2–4 IV then oral 8 mg IV day 1, then 4 mg orally twice daily days 2–4; OAOD + thalidomide	Olanzapine 5 mg oral once daily, days 1–4 + Placebo matching capsule oral once daily, days 1–5 + Aprepitant 125 mg on day 1, then 80 mg on days 2–3 oral once daily + Ondansetron 8 mg IV then oral 8 mg IV 30 min before chemotherapy on day 1, then 8 mg orally three times daily on days 2–4 + Dexamethasone 8 mg on day 1, then 4 mg on days 2–4 IV then oral 8 mg IV day 1, then 4 mg orally twice daily days 2–4; OAOD + placebo
Harikul et al. [25, 29]	140	Carboplatin-, oxaliplatin-, or irinotecan-based chemotherapy; Carboplatin AUC ≥ 5 IV day 1 + Oxaliplatin IV day 1 + Irinotecan IV day 1	Moderate	Olanzapine 5 mg PO day 1 before chemotherapy, then once daily at bedtime on days 2–4 + Ondansetron 8 mg IV 30 min before chemotherapy on day 1 + Ondansetron 8 mg PO Twice daily on days 2–4 + Dexamethasone 12 mg IV 30 min before chemotherapy on day 1; OLN5 + ondansetron + dexamethasone	Placebo matching capsule PO day 1 before chemotherapy, then once daily at bedtime on days 2–4 + Ondansetron 8 mg IV 30 min before chemotherapy on day 1 + Ondansetron 8 mg PO Twice daily on days 2–4 + Dexamethasone 12 mg IV 30 min before chemotherapy on day 1; Placebo + ondansetron + dexamethasone
Hashimoto et al. [30, 31]	710	Cisplatin-based chemotherapy; cisplatin IV day 1, first-line	High	Olanzapine 5 mg oral once daily after dinner on days 1–4 + aprepitant 125 mg on day 1; 80 mg on days 2–3 oral day 1 before cisplatin, then after breakfast on days 2–3 + fosaprepitant 150 mg IV day 1, at a maximum of 1 h before cisplatin; used as alternative to oral aprepitant + palonosetron 0.75 mg IV day 1, at a maximum of 30 min before cisplatin + dexamethasone 12 mg day 1; 8 mg days 2–4; if fosaprepitant used, 16 mg on days 3–4 oral or IV day 1 before cisplatin, then days 2–4; Olanzapine group	Placebo matching capsule oral once daily after dinner on days 1–4 + aprepitant 125 mg on day 1; 80 mg on days 2–3 oral day 1 before cisplatin, then after breakfast on days 2–3 + fosaprepitant 150 mg IV day 1, at a maximum of 1 h before cisplatin; used as alternative to oral aprepitant + palonosetron 0.75 mg IV day 1, at a maximum of 30 min before cisplatin + dexamethasone 12 mg day 1; 8 mg days 2–4; if fosaprepitant used, 16 mg on days 3–4 oral or IV day 1 before cisplatin, then days 2–4; Placebo group
Inui et al. [32]	378	Carboplatin-containing chemotherapy; Carboplatin IV first course, day 1	Moderate	Olanzapine 5 mg oral once daily on days 1–4 after the evening meal + Aprepitant 125 mg on day 1; 80 mg on days 2–3 oral once daily + 5-HT3 receptor antagonist palonosetron 0.75 mg IV or granisetron 2 mg PO/IV dose per package insert IV or PO day 1 + Dexamethasone 4.95 mg IV day 1; Olanzapine group	Placebo matching placebo oral once daily on days 1–4 after the evening meal + Aprepitant 125 mg on day 1; 80 mg on days 2–3 oral once daily + 5-HT3 receptor antagonist palonosetron 0.75 mg IV or granisetron 2 mg PO/IV dose per package insert IV or PO day 1 + Dexamethasone 4.95 mg IV day 1; Placebo group
Ithimakin et al. [33]	140	High-emetogenic chemotherapy: doxorubicin/cyclophosphamide or cisplatin-based therapy; Doxorubicin 60 mg/m^2^ IV day 1 + Cyclophosphamide 600 mg/m^2^ IV day 1 + Cisplatin > = 50 mg/m^2^ IV day 1	High	Olanzapine 5 mg oral day 1 before chemotherapy, then days 2–4 + Ondansetron 8 mg IV 30 min before chemotherapy on day 1 + Dexamethasone 12 mg IV day 1; 8 mg oral days 2–4 IV/oral day 1 before chemotherapy, then days 2–4; OLN5	Aprepitant 125 mg day 1; 80 mg days 2–3 oral day 1 before chemotherapy, then days 2–3 + Ondansetron 8 mg IV 30 min before chemotherapy on day 1 + Dexamethasone 12 mg IV day 1; 8 mg oral days 2–4 IV/oral day 1 before chemotherapy, then days 2–4; APR
Jeon et al. [34]	56	Moderately emetogenic chemotherapy	Moderate	Olanzapine 10 mg oral daily on days 1 through 4 + Palonosetron + Dexamethasone; Olanzapine arm	Placebo matching placebo oral daily on days 1 through 4 + Palonosetron + Dexamethasone; Placebo arm
Khani et al. [35]	73	Mixed highly or moderately emetogenic chemotherapy	Mixed	Olanzapine 5 mg (body weight ‚â§60 kg) or 10 mg (body weight > 60 kg) PO day before chemotherapy and continued for days 1–5 after chemotherapy + dexamethasone 8 mg day 1 of chemotherapy + granisetron 1 mg three times daily on day 1 of chemotherapy; OGD	Placebo PO day before chemotherapy and maintained for 1–5 days following chemotherapy + dexamethasone 8 mg day 1 of chemotherapy + granisetron 1 mg three times daily on day 1 of chemotherapy; PGD
Liu et al. [36, 37]	560	Cisplatin-based doublet highly emetogenic chemotherapy; Cisplatin IV	High	Olanzapine 5 mg + 5-HT3 receptor antagonist + NK-1 receptor antagonist; Olanzapine group	Dexamethasone + 5-HT3 receptor antagonist + NK-1 receptor antagonist; Dexamethasone group
Liu et al. [38]	222	Three-day cisplatin-based chemotherapy; cisplatin 25 mg/m^2^/day IV days 1–3	High	Olanzapine 5 mg PO days 1–4 + tropisetron 5 mg IV days 1–3 + dexamethasone 10 mg IV days 1–3; Olanzapine regimen	Aprepitant 125 mg PO day 1 + aprepitant 80 mg PO days 2–3 + tropisetron 5 mg IV days 1–3 + dexamethasone 5 mg IV days 1–3; Aprepitant regimen
Maleki et al. [39]	69	AC (doxorubicin/cyclophosphamide) adjuvant chemotherapy; Doxorubicin 50 mg/m^2^ day 1, cycle 1 and cycle 2 + Cyclophosphamide 500 mg/m^2^ day 1, cycle 1 and cycle 2	High	Olanzapine 10 mg (5 mg if age ≥60 years) oral days 1–4 after chemotherapy + Aprepitant 125 mg on day 1, then 80 mg on days 2–3 oral day 1; days 2–3 + Dexamethasone 12 mg IV day 1, 30–60 min before chemotherapy + Granisetron 1 mg IV day 1, 30–60 min before chemotherapy; ADG-O	Aprepitant 125 mg on day 1, then 80 mg on days 2–3 oral day 1; days 2–3 + Dexamethasone 12 mg IV day 1, 30–60 min before chemotherapy + Granisetron 1 mg IV day 1, 30–60 min before chemotherapy + Mirtazapine 15 mg oral days 1–4 after chemotherapy; ADG-M
Minatogawa et al. [40]	278	Cisplatin-based chemotherapy; Cisplatin‚ ≥50 mg/m^2^ IV first-course cisplatin-based chemotherapy; day 1	High	Olanzapine 5 mg PO days 1–4 after dinner + Palonosetron 0.75 mg IV infusion day 1, 30 min before cisplatin + NK1-RA (aprepitant or fosaprepitant) Aprepitant 125 mg day 1 and 80 mg days 2–3, or fosaprepitant 150 mg day 1 PO or IV day 1 before cisplatin; aprepitant continues days 2–3 + Dexamethasone 9.9 mg day 1; 6.6 mg days 2–4 IV (13.2 mg on days 3–4 if fosaprepitant used) IV infusion days 1–4; Arm D4	Olanzapine 5 mg PO days 1–4 after dinner + Palonosetron 0.75 mg IV infusion day 1, 30 min before cisplatin + NK1-RA (aprepitant or fosaprepitant) Aprepitant 125 mg day 1 and 80 mg days 2–3, or fosaprepitant 150 mg day 1 PO or IV day 1 before cisplatin; aprepitant continues days 2–3 + Dexamethasone 9.9 mg IV infusion day 1 only + Placebo IV days 2–4; Arm D1
Mizukami et al. [41]	48	Mixed highly or moderately emetogenic chemotherapy; cisplatin IV + epirubicin IV + cyclophosphamide IV + doxorubicin IV + nedaplatin IV + carboplatin IV + daunorubicin IV	Mixed	Olanzapine 5 mg/day oral day 0 to day 5 + dexamethasone 9.9 mg on day 1, then 6.6 mg on days 2–4 IV days 1–4 + 5-HT3 receptor antagonist granisetron 3–6 mg/day days 1–3, or ondansetron 4 mg/day days 1–2, or ramosetron 0.6 mg/day days 1–3, or palonosetron 0.75 mg Day 1 IV Days 1–3 depending on agent + aprepitant 125 mg on Day 1, then 80 mg on Days 2–3 oral Days 1–3; OL group	Placebo daily oral day 0 to Day 5 + dexamethasone 9.9 mg on day 1, then 6.6 mg on days 2–4 IV days 1–4 + 5-HT3 receptor antagonist granisetron 3–6 mg/day days 1–3, or ondansetron 4 mg/day days 1–2, or ramosetron 0.6 mg/day days 1–3, or palonosetron 0.75 mg Day 1 IV days 1–3 depending on agent + aprepitant 125 mg on day 1, then 80 mg on days 2–3 oral days 1–3; Control group
Mohammed et al. [42]	154	Highly emetogenic chemotherapy; cisplatin + doxorubicin + cyclophosphamide	High	Olanzapine 5 mg once daily on days 1–4 + palonosetron + dexamethasone; Olanzapine triple therapy	Palonosetron + dexamethasone + aprepitant 125 mg on day 1, 80 mg on days 2–3 days 1–3 + fosaprepitant 150 mg IV day 1; Aprepitant/fosaprepitant triple therapy
Mukhopadhyay et al. [43]	100	Single-day platinum-based chemotherapy; Cisplatin high-dose considered HEC; lower dose considered MEC IV day 1, single-day cycle + Carboplatin IV day 1, single-day cycle + Oxaliplatin IV day 1, single-day cycle	Mixed	Olanzapine 10 mg oral day 1 through day 5, once daily + Palonosetron 0.25 mg IV day 1, 30–60 min before chemotherapy + Dexamethasone 8 mg IV for MEC or 16 mg IV for HEC on day 1; then 8 mg PO daily on days 2–3 for MEC or 8 mg PO twice daily on days 2–4 for HEC IV day 1; oral days 2–4 30–60 min before chemotherapy on day 1, then post-chemotherapy continuation by emetogenicity; Test group	Palonosetron 0.25 mg IV day 1, 30–60 min before chemotherapy + Dexamethasone 8 mg IV for MEC or 16 mg IV for HEC on day 1; then 8 mg PO daily on days 2–3 for MEC or 8 mg PO twice daily on days 2–4 for HEC IV day 1; oral days 2–4 30–60 min before chemotherapy on day 1, then post-chemotherapy continuation by emetogenicity; Control group
Mukhopadhyay et al. [44]	58	Single-day high and moderately emetogenic chemotherapy	Mixed	Olanzapine 10 mg oral days 1–3 + Ondansetron 16 mg day 1 + Dexamethasone 8 mg days 1–3; Control	Olanzapine 5 mg oral days 1–3 + Ondansetron 16 mg day 1 + Dexamethasone 8 mg days 1–3; Test
Nakashima et al. [45]	69	Moderately emetogenic chemotherapy	Moderate	Olanzapine 10 mg days 1–3; daytime or evening + Ondansetron + Dexamethasone; Control group	Olanzapine 5 mg days 1–3; daytime or evening + Ondansetron + Dexamethasone; Test group
Navari et al. [46]	247	Highly emetogenic chemotherapy: cisplatin or doxorubicin/cyclophosphamide; Cisplatin + Doxorubicin + Cyclophosphamide	High	Olanzapine 10 mg PO day 1 prechemotherapy, then 10 mg daily on days 2–4 + Palonosetron 0.25 mg IV day 1, 30–60 min prior to chemotherapy + Dexamethasone 20 mg IV day 1, 30–60 min prior to chemotherapy; OPD	Aprepitant 125 mg PO day 1, 30–60 min prior to chemotherapy; then 80 mg/day PO on days 2–3 + Palonosetron 0.25 mg IV day 1, 30–60 min prior to chemotherapy + Dexamethasone 12 mg IV day 1; 4 mg BID days 2–4 IV/PO day 1 prechemotherapy, then PO BID on days 2–4; APD
Navari et al. [47]	118	Concurrent cisplatin plus 5-fluorouracil with radiotherapy; cisplatin > 70 mg/m2 IV day 1 concurrent with radiotherapy + 5-fluorouracil 750 mg/m^2^/day IV daily for 4 days concurrent with radiotherapy	High	Olanzapine 10 mg PO day 1 before chemotherapy, then 10 mg PO daily on days 2–4 + palonosetron 0.25 mg IV day 1, 30–60 min before chemotherapy + dexamethasone 20 mg IV day 1, 30–60 min before chemotherapy; OPD	Fosaprepitant 150 mg IV day 1, 30–60 min before chemotherapy + palonosetron 0.25 mg IV day 1, 30–60 min before chemotherapy + dexamethasone 12 mg IV day 1, 30–60 min before chemotherapy + dexamethasone 4 mg BID PO days 2–3; FPD
Navari et al. [3]	401	Highly emetogenic chemotherapy: cisplatin-containing regimen or doxorubicin/cyclophosphamide; Cisplatin 70 mg/m^2^ day 1 + Doxorubicin 60 mg/m^2^ day 1 + Cyclophosphamide 600 mg/m^2^ day 1	High	Olanzapine 10 mg oral days 1–4 + Dexamethasone 12 mg on day 1; 8 mg on days 2–4 oral days 1–4 + Aprepitant or fosaprepitant Aprepitant 125 mg on day 1 and 80 mg on days 2–3, oral; or fosaprepitant 150 mg on day 1, IV oral or IV day 1 with optional days 2–3 for aprepitant + 5-HT3 receptor antagonist Palonosetron 0.25 mg IV, or granisetron 1 mg IV/2 mg oral, or ondansetron 8 mg IV or oral IV or oral day 1	Placebo matching placebo oral days 1–4 + Dexamethasone 12 mg on day 1; 8 mg on days 2–4 oral days 1–4 + Aprepitant or fosaprepitant Aprepitant 125 mg on day 1 and 80 mg on days 2–3, oral; or fosaprepitant 150 mg on day 1, IV oral or IV day 1 with optional days 2–3 for aprepitant + 5-HT3 receptor antagonist Palonosetron 0.25 mg IV, or granisetron 1 mg IV/2 mg oral, or ondansetron 8 mg IV or oral IV or oral day 1
Navari et al. [48]	690	Highly emetogenic intravenous chemotherapy; cisplatin ≥ 70 mg/m^2^ IV single day, day 1 + doxorubicin 60 mg/m^2^ IV day 1 + cyclophosphamide 600 mg/m^2^ IV day 1	High	Olanzapine 10 mg/day PO days 1–4; prior to chemotherapy or at bedtime on day 1 + dexamethasone 12 mg day 1, then 8 mg days 2–4 PO day 1 prior to chemotherapy; days 2–4 + palonosetron or ondansetron palonosetron 0.25 mg IV or ondansetron 8–16 mg IV or 16–24 mg PO IV or PO day 1 prior to chemotherapy + fosaprepitant or aprepitant fosaprepitant 150 mg IV or aprepitant 130 mg IV IV day 1 prior to chemotherapy; Olanzapine + 5-HT3 RA + dexamethasone + NK-1 RA	Olanzapine 10 mg/day PO days 1–4, approximately every 24 h + placebo day 1 prior to chemotherapy + 5-HT3 receptor antagonist palonosetron 0.25 mg IV or ondansetron 8–16 mg IV or 16–24 mg PO IV or PO day 1 prior to chemotherapy + dexamethasone 12 mg PO day 1; 8 mg PO days 2–4 PO days 1–4; Placebo arm
Ostwal et al. [49]	560	Mixed moderately emetogenic chemotherapy regimens	Moderate	Olanzapine 10 mg oral once at night on days 1–3 + Dexamethasone 12 mg IV day 1 + Aprepitant 125 mg on day 1; 80 mg on days 2–3 oral day 1, then days 2–3 + Palonosetron 0.25 mg IV day 1; Olanzapine group	Dexamethasone 12 mg IV day 1 + Aprepitant 125 mg on day 1; 80 mg on days 2–3 oral day 1, then days 2–3 + Palonosetron 0.25 mg IV day 1; Observation group
Prasad et al. [50]	50	Highly emetogenic chemotherapy	High	Olanzapine 10 mg PO day 1, then once daily for next 3 days + Palonosetron 0.25 mg IV day 1 + Dexamethasone 20 mg IV day 1; OPD	Aprepitant 125 mg PO day 1 + Aprepitant 80 mg PO once daily on days 2 and 3 + Palonosetron 0.25 mg IV day 1 + Dexamethasone 12 mg IV day 1 + Dexamethasone 8 mg PO once daily on days 2 and 3 + Dexamethasone 8 mg PO once on day 4; APD
Radhakrishnan et al. [51]	350	Single-day highly emetogenic chemotherapy; doxorubicin (adriamycin) + cyclophosphamide + cisplatin	Mixed	Olanzapine 5 mg oral 30 min before chemotherapy on day 1, then once daily on days 2–4 starting 24 h after first dose + Palonosetron 0.25 mg IV 30 min before chemotherapy on day 1 + Fosaprepitant 150 mg IV infusion day 1 in 150 ml 0.9% saline over 30 min, completing 30 min before chemotherapy; OPF	Olanzapine 5 mg oral 30 min before chemotherapy on day 1, then once daily on days 2–4 starting 24 h after first dose + Palonosetron 0.25 mg IV 30 min before chemotherapy on day 1 + Dexamethasone 12 mg IV infusion day 1 in 150 ml 0.9% saline over 30 min, completing 30 min before chemotherapy; OPD
Ramasubbu et al. [52]	54	Mixed highly emetogenic chemotherapy regimens	Mixed	Olanzapine 5 mg oral day 1 with chemotherapy, then once nightly on days 2–5 + Pregabalin 75 mg oral day 1 with chemotherapy, then once nightly on days 2–5 + Ondansetron 8 mg IV day 1, 30 min before chemotherapy + Dexamethasone 12 mg IV day 1, 30 min before chemotherapy + Dexamethasone 8 mg oral days 2–4; Standard of care + olanzapine 5 mg + pregabalin 75 mg	Placebo oral day 1 with chemotherapy, then once nightly on days 2–5 + Ondansetron 8 mg IV day 1, 30 min before chemotherapy + Dexamethasone 12 mg IV day 1, 30 min before chemotherapy + Dexamethasone 8 mg oral days 2–4; Standard of care + placebo
Rumyantsev et al. [53]	93	Cisplatin-based, AC, and other regimens; cisplatin + carboplatin + doxorubicin + cyclophosphamide	Mixed	Olanzapine 5 mg QD days 0–4 + ondansetron 16 mg day 1 + dexamethasone 8 mg days 1–3; OLN	Aprepitant 125 mg day 1 + aprepitant 80 mg days 2–3 + ondansetron 16 mg day 1 + dexamethasone 8 mg days 1–3; APR
Saito et al. [54]	500	Anthracycline plus cyclophosphamide-based chemotherapy for breast cancer	High	Olanzapine 5 mg oral within 5 h after chemotherapy and before the evening meal on day 1; after the evening meal on days 2–4 + Palonosetron 0.75 mg IV day 1, 30 min before chemotherapy + Aprepitant 125 mg on day 1, then 80 mg on days 2–3 oral day 1, 1 h before chemotherapy; days 2–3 in the morning + Fosaprepitant 150 mg IV day 1, 1 h before chemotherapy, used instead of oral aprepitant per hospital policy + Dexamethasone 9.9 mg IV day 1, 30 min before chemotherapy; Olanzapine group	Placebo oral Within 5 h after chemotherapy and before the evening meal on day 1; after the evening meal on days 2–4 + Palonosetron 0.75 mg IV day 1, 30 min before chemotherapy + Aprepitant 125 mg on day 1, then 80 mg on days 2–3 oral day 1, 1 h before chemotherapy; days 2–3 in the morning + Fosaprepitant 150 mg IV day 1, 1 h before chemotherapy, used instead of oral aprepitant per hospital policy + Dexamethasone 9.9 mg IV day 1, 30 min before chemotherapy; Placebo group
Sakai et al. [55]	168	Trastuzumab deruxtecan (T-DXd); trastuzumab deruxtecan first cycle	Moderate	Olanzapine 5 mg oral once daily, days 1–6 + 5-HT3 receptor antagonist + dexamethasone 6.6 mg IV or 8 mg oral IV or oral day 1; OLZ	Placebo oral once daily, days 1–6 + 5-HT3 receptor antagonist + dexamethasone 6.6 mg IV or 8 mg oral IV or oral day 1; PBO
Saldanha et al. [56, 57]	209	Highly emetogenic chemotherapy; cisplatin + cyclophosphamide + doxorubicin	High	Olanzapine 10 mg oral daily on days 1–4 + aprepitant standard dose before and after chemotherapy + dexamethasone standard dose before and after chemotherapy + 5-HT3 antagonist standard dose before and after chemotherapy; Combined (AHDO)	Aprepitant standard dose before and after chemotherapy + dexamethasone standard dose before and after chemotherapy + 5-HT3 antagonist standard dose before and after chemotherapy; Standard (AHD)
Sapkota et al. [58]	50	Highly emetogenic chemotherapy protocols; Cisplatin > 60 mg/m^2^ + Doxorubicin (Adriamycin) > 60 mg/m^2^ + Epirubicin > 90 mg/m^2^ + Ifosfamide > 2 mg/m^2^	High	Olanzapine 10 mg PO Day 1 and days 2–4 + Granisetron 1 mg IV day 1 + Dexamethasone 12 mg on day 1; 8 mg PO 12 mg day 1, then 8 mg once daily on days 2–4; Arm B	Aprepitant 180 mg on day 1; 80 mg PO 180 mg day 1, then 80 mg on days 2–3 + Granisetron 1 mg IV day 1 + Dexamethasone 12 mg on day 1; 8 mg PO 12 mg day 1, then 8 mg once daily on days 2–4; Arm A
Shanthilal et al. [59, 60]	141	AC; Doxorubicin 60 mg/m^2^ + Cyclophosphamide 600 mg/m^2^	High	Olanzapine 10 mg oral day 1 + Aprepitant 125 mg day 1; 80 mg days 2–3 oral days 1–3 + Palonosetron 0.25 mg day 1 + Dexamethasone 8 mg day 1; Combination	Aprepitant 125 mg day 1; 80 mg days 2–3 oral days 1–3 + Palonosetron 0.25 mg day 1 + Dexamethasone 8 mg day 1
Sharma et al. [61]	280	Highly emetogenic chemotherapy	High	Olanzapine 10 mg + Palonosetron or granisetron + Dexamethasone; O10PD	Olanzapine 5 mg + Palonosetron or granisetron + Dexamethasone; O5PD
Shumway et al. [62]	18	Mixed highly emetogenic chemotherapy regimens	Mixed	Olanzapine 5 mg PO D-2 and D-1 + Olanzapine 10 mg PO D1-D4 + Dexamethasone (Decadron) 12 mg IV on D1; 4 mg PO BID on D2-D4 IV/PO D1-D4 + Palonosetron 0.25 mg IV D1; Experimental arm	Aprepitant 125 mg PO D1 + Dexamethasone (Decadron) 12 mg IV on D1; 4 mg PO BID on D2-D4 IV/PO D1-D4 + Palonosetron 0.25 mg IV D1 + Aprepitant 80 mg PO D2-D3; Standard arm
Sukauichai et al. [63]	140	AC or high-dose cisplatin-based chemotherapy; Doxorubicin 60 mg/m^2^ IV day 1 as part of AC + Cyclophosphamide 600 mg/m^2^ IV day 1 as part of AC + Cisplatin ≥ 70 mg/m^2^ IV day 1 + Fluorouracil may be given concurrently with cisplatin; unspecified + Etoposide may be given concurrently with cisplatin; unspecified	High	Olanzapine 10 mg oral after dinner, days 1–4 + Dexamethasone 20 mg IV 30 min before chemotherapy on day 1 + Dexamethasone 8 mg oral days 2–4 + Ondansetron 8 mg IV 30 min before chemotherapy on day 1 + Ondansetron 8 mg oral morning of day 2; Olanzapine 10 mg + ondansetron + dexamethasone	Olanzapine 5 mg oral after dinner, days 1–4 + Dexamethasone 20 mg IV 30 min before chemotherapy on day 1 + Dexamethasone 8 mg oral days 2–4 + Ondansetron 8 mg IV 30 min before chemotherapy on day 1 + Ondansetron 8 mg oral morning of day 2; Olanzapine 5 mg + ondansetron + dexamethasone
Tan et al. [64, 65]	229	Mixed highly and moderately emetogenic chemotherapy; cisplatin 75 mg/m^2^ + oxaliplatin 85 mg/m^2^ + epirubicin 90 mg/m^2^ + carboplatin AUC 5 + doxorubicin (adriamycin) 50 mg/m^2^ + dacarbazine 200 mg/m^2^	High	Olanzapine 10 mg PO day 1, then once daily on days 2–5 + azasetron 10 mg IV day 1 + dexamethasone 10 mg IV day 1; Test group—HEC subgroup	Azasetron 10 mg IV day 1 + dexamethasone 10 mg IV day 1, then once daily on days 2–5; Control group—HEC subgroup
Tranh et al. 2017	478	Dose-dense AC or FEC adjuvant chemotherapy	High	Olanzapine 10 mg oral once daily for 3 days + Omeprazole 20 mg oral once daily for 3 days; OO	Dexamethasone 4 mg oral twice daily for 5 days + Omeprazole 20 mg oral twice daily for 5 days + Metoclopramide 20 mg oral three times daily for 5 days; dexomeprim
Tienchaiananda et al. [66]	40	AC regimen; Doxorubicin 60 mg/m^2^ IV day 1 + Cyclophosphamide 600 mg/m^2^ IV day 1	High	Olanzapine 10 mg oral day 1 before chemotherapy, then once daily on days 2–4 + Ondansetron 8 mg IV 30 min before chemotherapy on day 1 + Dexamethasone 20 mg IV 30 min before chemotherapy on day 1 + Dexamethasone 10 mg/day oral days 2–4; Olanzapine group	Ondansetron 8 mg IV 30 min before chemotherapy on day 1 + Dexamethasone 20 mg IV 30 min before chemotherapy on day 1 + Dexamethasone 10 mg/day oral days 2–4; Placebo group
Tvsvgk et al. [67]	118	AC or EC; Doxorubicin + Epirubicin + Cyclophosphamide	High	Olanzapine 10 mg; Olanzapine 10 mg + standard antiemetic regimen	Olanzapine 5 mg; Olanzapine 5 mg + standard antiemetic regimen
Uppal et al. [68]	59	Carboplatin plus paclitaxel; carboplatin every 3 weeks + paclitaxel every 3 weeks; q21 days	Moderate	Olanzapine 5 mg PO nightly for 4 days starting day of chemotherapy + dexamethasone 20 mg IV day 1 prior to chemotherapy + ondansetron 8 mg IV or 16 mg PO IV or PO day 1 prior to chemotherapy + ondansetron 8 mg PO twice daily on days 2–4 after chemotherapy; Olanzapine	Fosaprepitant 150 mg IV single dose on day 1 prior to infusion + dexamethasone 20 mg IV day 1 prior to chemotherapy + ondansetron 8 mg IV or 16 mg PO IV or PO day 1 prior to chemotherapy + ondansetron 8 mg PO twice daily on days 2–4 after chemotherapy; Fosaprepitant
Vinayanuwattikun et al. [69]	100	High-dose cisplatin regimens; Cisplatin 75 mg/m^2^; median dose 125 mg IV	High	Olanzapine 10 mg/day PO days 1–4 + Netupitant/palonosetron (NEPA) netupitant 300 mg + palonosetron 0.5 mg PO day 1 of each cycle + Dexamethasone 10 mg IV day 1, then 4 mg PO once daily days 2–4 initially; after amendment 4 mg PO twice daily days 2–4 IV/PO Day 1 then days 2–4; Upfront netupitant strategy	Olanzapine 10 mg/day PO Days 1–4 each cycle + Ondansetron 8 mg IV Day 1, then 8 mg PO once daily days 2–4 IV/PO Cycle 1; continued in cycle 2 if complete response in cycle 1 + Dexamethasone 20 mg IV day 1, then 4 mg PO twice daily days 2–4 IV/PO Cycle 1; also used with crossover regimen + Netupitant/palonosetron (NEPA) netupitant 300 mg + palonosetron 0.5 mg PO Cycle 2 only if no complete response in cycle 1; Subsequent netupitant strategy
Wang et al. [70]	110	Three-day cisplatin-based double regimen; cisplatin 25 mg/m^2^/day IV days 1–3 + gemcitabine + docetaxel + etoposide + pemetrexed + paclitaxel + capecitabine + irinotecan + bevacizumab some patients also received	High	Olanzapine 5 mg PO once daily on days 1–3 or delayed to days 2–4 + fosaprepitant 150 mg IV day 1 or delayed to day 2 + palonosetron 0.25 mg IV day 1 + dexamethasone 5 mg IV days 1–3 (routine) or 10 mg day 1 then 5 mg days 2–3 (delayed) IV days 1–3; Quadruple regimen (pooled A + B)	Olanzapine 5 mg PO once daily on days 1–3 or delayed to days 2–4 + palonosetron 0.25 mg IV day 1 + dexamethasone 10 mg IV once daily on days 1–3; Triple regimen (pooled C + D)
Zhang et al. [71]	77	5-day cisplatin-based chemotherapy (BEP, VIP, or EP); cisplatin 20 mg/m^2^/day days 1–5 + bleomycin + etoposide + ifosfamide	High	Olanzapine 5 mg oral nightly, days 1–7 + ondansetron hydrochloride 8 mg IV 30 min before chemotherapy on days 1–5 + fosaprepitant 150 mg IV 1 h before chemotherapy on days 1 and 4 + dexamethasone 6 mg oral 30 min before chemotherapy on days 1–7; Olanzapine + standard antiemetics	Placebo matching placebo oral nightly, days 1–7 + ondansetron hydrochloride 8 mg IV 30 min before chemotherapy on days 1–5 + fosaprepitant 150 mg IV 1 h before chemotherapy on days 1 and 4 + dexamethasone 6 mg oral 30 min before chemotherapy on days 1–7; Placebo + standard antiemetics
Zhao et al. [72]	349	Multiday cisplatin-containing chemotherapy; cisplatin 3-day total dose 75 mg/m^2^ IV days 1–3	High	Olanzapine 5 mg oral before bedtime, days 1–5 + fosaprepitant 150 mg IV approximately 1 h before cisplatin on day 1 + ondansetron hydrochloride 8 mg IV 30 min before cisplatin on days 1–3 + dexamethasone 6 mg oral 30 min before cisplatin on days 1–3; also on days 4–5; Olanzapine + triple antiemetic regimen	Placebo matching placebo oral before bedtime, days 1–5 + fosaprepitant 150 mg IV approximately 1 h before cisplatin on day 1 + ondansetron hydrochloride 8 mg IV 30 min before cisplatin on days 1–3 + dexamethasone 6 mg oral 30 min before cisplatin on days 1–3; also on days 4–5; Placebo + triple antiemetic regimen

## Appendix 3

See Table 3

**Table 3:** Complete response (odds ratio and 95% confidence intervals), subgroup analysis of highly emetogenic chemotherapy patients. Odds ratios > 1 favor the row-defining treatment

3.1 Acute phase					
	Placebo	OLN10	OLN5	OLN2.5	Other
Placebo	—	0.46 (0.23–0.91)	0.50 (0.25–0.98)	0.70 (0.27–1.81)	0.62 (0.29–1.31)
OLN10	2.17 (1.10–4.35)	—	1.09 (0.52–2.27)	1.52 (0.54–4.35)	1.35 (0.57–3.23)
OLN5	2.00 (1.02–4.00)	0.92 (0.44–1.92)	—	1.39 (0.51–3.85)	1.24 (0.53–2.94)
OLN2.5	1.43 (0.55–3.70)	0.66 (0.23–1.85)	0.72 (0.26–1.96)	—	0.89 (0.28–2.86)
Other	1.61 (0.76–3.45)	0.74 (0.31–1.75)	0.81 (0.34–1.89)	1.12 (0.35–3.57)	—
3.2 Delayed phase					
	Placebo	OLN10	OLN5	OLN2.5	Other
Placebo	—	0.39 (0.19–0.78)	0.43 (0.22–0.84)	0.62 (0.25–1.56)	0.56 (0.27–1.18)
OLN10	2.56 (1.28–5.26)	—	1.09 (0.52–2.27)	1.57 (0.57–4.35)	1.43 (0.61–3.33)
OLN5	2.33 (1.19–4.55)	0.92 (0.44–1.92)	—	1.44 (0.53–3.92)	1.31 (0.57–3.03)
OLN2.5	1.61 (0.64–4.00)	0.64 (0.23–1.75)	0.69 (0.26–1.89)	—	0.91 (0.29–2.86)
Other	1.79 (0.85–3.70)	0.70 (0.30–1.64)	0.76 (0.33–1.75)	1.10 (0.35–3.45)	—
3.3 Long-delayed					
	Placebo	OLN5			
Placebo	—	0.46 (0.30–0.71)			
OLN5	2.17 (1.41–3.33)	—			
3.4 Overall phase					
	Placebo	OLN10	OLN5	OLN2.5	Other
Placebo	—	0.45 (0.29–0.71)	0.50 (0.32–0.77)	0.71 (0.31–1.61)	0.59 (0.32–1.09)
OLN10	2.22 (1.41–3.45)	—	1.12 (0.67–1.85)	1.58 (0.67–3.70)	1.31 (0.67–2.56)
OLN5	2.00 (1.30–3.13)	0.89 (0.54–1.49)	—	1.41 (0.61–3.23)	1.17 (0.61–2.27)
OLN2.5	1.41 (0.62–3.23)	0.63 (0.27–1.49)	0.71 (0.31–1.64)	—	0.83 (0.29–2.38)
Other	1.69 (0.92–3.13)	0.76 (0.39–1.49)	0.85 (0.44–1.64)	1.20 (0.42–3.45)	—

## Appendix 4

See Table 4

**Table 4:** Complete response (odds ratio and 95% confidence intervals), subgroup analysis of moderately emetogenic chemotherapy patients. Odds ratios > 1 favor the row-defining treatment

4.1 Acute phase			
	Placebo	OLN10	OLN5
Placebo	—	0.58 (0.24–1.41)	0.68 (0.46–0.98)
OLN10	1.72 (0.71–4.17)	—	1.18 (0.52–2.70)
OLN5	1.47 (1.02–2.17)	0.85 (0.37–1.92)	—
4.2 Delayed phase			
	Placebo	OLN10	OLN5
Placebo	—	0.46 (0.21–1.00)	0.68 (0.50–0.93)
OLN10	2.17 (1.00–4.76)	—	1.47 (0.76–2.86)
OLN5	1.47 (1.08–2.00)	0.68 (0.35–1.31)	—
4.3 Long-delayed			
	Placebo	OLN5	
Placebo	—	0.46 (0.30–0.71)	
OLN5	2.17 (1.41–3.33)	—	
4.4 Overall phase			
	Placebo	OLN10	OLN5
Placebo	—	0.58 (0.28–1.19)	0.68 (0.50–0.93)
OLN10	1.72 (0.84–3.57)	—	1.47 (0.76–2.86)
OLN5	1.47 (1.08–2.00)	0.68 (0.35–1.31)	—

## Appendix 5

See Table 5

**Table 5:** Complete control (odds ratio and 95% confidence intervals). Odds ratios > 1 favor the row-defining treatment

5.1 Acute phase					
	Placebo	OLN10	OLN5	OLN2.5	Other
Placebo	—	0.49 (0.29–0.88)	0.71 (0.52–0.97)	0.67 (0.39–1.15)	0.86 (0.49–1.51)
OLN10	2.04 (1.14–3.45)	—	1.45 (0.83–2.52)	1.36 (0.69–2.67)	1.76 (0.85–3.63)
OLN5	1.41 (1.03–1.92)	0.69 (0.40–1.20)	—	0.94 (0.50–1.77)	1.21 (0.62–2.35)
OLN2.5	1.49 (0.87–2.56)	0.74 (0.37–1.45)	1.06 (0.57–2.00)	—	1.29 (0.58–2.86)
Other	1.16 (0.66–2.04)	0.57 (0.28–1.18)	0.83 (0.43–1.62)	0.78 (0.35–1.72)	—
5.2 Delayed phase					
	Placebo	OLN10	OLN5	OLN2.5	Other
Placebo	—	0.40 (0.24–0.68)	0.66 (0.48–0.91)	0.59 (0.37–0.95)	0.73 (0.45–1.18)
OLN10	2.50 (1.47–4.17)	—	1.65 (0.96–2.83)	1.47 (0.78–2.77)	1.83 (0.95–3.53)
OLN5	1.52 (1.10–2.08)	0.61 (0.35–1.04)	—	0.89 (0.52–1.54)	1.11 (0.65–1.90)
OLN2.5	1.69 (1.05–2.70)	0.68 (0.36–1.28)	1.12 (0.65–1.92)	—	1.24 (0.66–2.35)
Other	1.37 (0.85–2.22)	0.55 (0.28–1.05)	0.90 (0.53–1.55)	0.81 (0.43–1.51)	—
5.3 Long-delayed phase					
	Placebo	OLN5			
Placebo	—	0.36 (0.23–0.58)			
OLN5	2.78 (1.72–4.35)	—			
5.4 Overall phase					
	Placebo	OLN10	OLN5	OLN2.5	Other
Placebo	—	0.49 (0.32–0.77)	0.72 (0.53–0.98)	0.61 (0.40–0.94)	0.67 (0.45–1.00)
OLN10	2.04 (1.30–3.13)	—	1.47 (1.00–2.16)	1.25 (0.82–1.90)	1.37 (0.90–2.08)
OLN5	1.39 (1.02–1.89)	0.68 (0.46–1.00)	—	0.85 (0.58–1.25)	0.93 (0.64–1.36)
OLN2.5	1.64 (1.06–2.50)	0.80 (0.53–1.22)	1.18 (0.80–1.73)	—	1.10 (0.71–1.70)
Other	1.49 (1.00–2.22)	0.73 (0.48–1.11)	1.08 (0.74–1.57)	0.91 (0.59–1.41)	—

## Appendix 6

See Table 6

**Table 6:** Complete control (odds ratio and 95% confidence intervals), subgroup analysis of highly emetogenic chemotherapy patients. Odds ratios > 1 favor the row-defining treatment

6.1 Acute phase					
	Placebo	OLN10	OLN5	OLN2.5	Other
Placebo	—	0.44 (0.24–0.81)	0.52 (0.31–0.88)	0.77 (0.33–1.79)	0.67 (0.34–1.35)
OLN10	2.27 (1.23–4.17)	—	1.18 (0.63–2.22)	1.75 (0.69–4.35)	1.52 (0.71–3.23)
OLN5	1.92 (1.14–3.23)	0.85 (0.45–1.59)	—	1.48 (0.60–3.57)	1.29 (0.61–2.70)
OLN2.5	1.30 (0.56–3.03)	0.57 (0.23–1.45)	0.68 (0.28–1.67)	—	0.87 (0.31–2.50)
Other	1.49 (0.74–2.94)	0.66 (0.31–1.41)	0.77 (0.37–1.64)	1.15 (0.40–3.23)	—
6.2 Delayed phase					
	Placebo	OLN10	OLN5	OLN2.5	Other
Placebo	—	0.39 (0.22–0.69)	0.48 (0.29–0.79)	0.68 (0.31–1.49)	0.61 (0.31–1.19)
OLN10	2.56 (1.45–4.55)	—	1.23 (0.67–2.27)	1.75 (0.73–4.17)	1.56 (0.74–3.23)
OLN5	2.08 (1.27–3.45)	0.81 (0.44–1.49)	—	1.43 (0.60–3.33)	1.27 (0.61–2.63)
OLN2.5	1.47 (0.67–3.23)	0.57 (0.24–1.37)	0.70 (0.30–1.67)	—	0.89 (0.33–2.38)
Other	1.64 (0.84–3.23)	0.64 (0.31–1.35)	0.79 (0.38–1.64)	1.12 (0.42–3.03)	—
6.3 Long-delayed phase					
	Placebo	OLN5			
Placebo	—	0.36 (0.23–0.58)			
OLN5	2.78 (1.72–4.35)	—			
6.4 Overall phase					
	Placebo	OLN10	OLN5	OLN2.5	Other
Placebo	—	0.43 (0.27–0.69)	0.50 (0.32–0.79)	0.74 (0.34–1.61)	0.63 (0.33–1.19)
OLN10	2.33 (1.45–3.70)	—	1.17 (0.72–1.92)	1.72 (0.75–3.92)	1.46 (0.75–2.86)
OLN5	2.00 (1.27–3.13)	0.85 (0.52–1.39)	—	1.47 (0.64–3.33)	1.25 (0.64–2.44)
OLN2.5	1.35 (0.62–2.94)	0.58 (0.26–1.33)	0.68 (0.30–1.56)	—	0.85 (0.32–2.27)
Other	1.59 (0.84–3.03)	0.68 (0.35–1.33)	0.80 (0.41–1.56)	1.18 (0.44–3.13)	—

## Appendix 7

See Table 7

**Table 7:** Complete control (odds ratio and 95% confidence intervals), subgroup analysis of moderately emetogenic chemotherapy patients. Odds ratios > 1 favor the row-defining treatment

7.1 Acute phase			
	Placebo	OLN10	OLN5
Placebo	—	0.58 (0.27–1.25)	0.68 (0.48–0.95)
OLN10	1.72 (0.80–3.70)	—	1.18 (0.58–2.44)
OLN5	1.47 (1.05–2.08)	0.85 (0.41–1.72)	—
7.2 Delayed phase			
	Placebo	OLN10	OLN5
Placebo	—	0.46 (0.23–0.93)	0.68 (0.49–0.93)
OLN10	2.17 (1.08–4.35)	—	1.47 (0.76–2.78)
OLN5	1.47 (1.08–2.04)	0.68 (0.36–1.31)	—
7.3 Long-Delayed Phase			
	Placebo	OLN5	
Placebo	—	0.36 (0.23–0.58)	
OLN5	2.78 (1.72–4.35)	—	
7.4 Overall Phase			
	Placebo	OLN10	OLN5
Placebo	—	0.58 (0.28–1.19)	0.68 (0.50–0.93)
OLN10	1.72 (0.84–3.57)	—	1.47 (0.76–2.86)
OLN5	1.47 (1.08–2.00)	0.68 (0.35–1.31)	—

## Appendix 8

See Table 8

**Table 8:** No nausea (odds ratio and 95% confidence intervals). Odds ratios > 1 favor the row-defining treatment

8.1 Acute phase					
	Placebo	OLN10	OLN5	OLN2.5	Other
Placebo	—	0.49 (0.24–1.02)	0.71 (0.51–0.99)	0.69 (0.39–1.22)	0.83 (0.45–1.52)
OLN10	2.04 (0.98–4.17)	—	1.45 (0.70–3.01)	1.40 (0.60–3.28)	1.69 (0.69–4.12)
OLN5	1.41 (1.01–1.96)	0.69 (0.33–1.43)	—	0.97 (0.48–1.95)	1.16 (0.57–2.38)
OLN2.5	1.45 (0.82–2.56)	0.71 (0.31–1.67)	1.03 (0.51–2.08)	—	1.20 (0.52–2.76)
Other	1.20 (0.66–2.22)	0.59 (0.24–1.44)	0.86 (0.42–1.75)	0.83 (0.36–1.94)	—
8.2 Delayed phase					
	Placebo	OLN10	OLN5	OLN2.5	Other
Placebo	—	0.40 (0.24–0.67)	0.51 (0.37–0.71)	0.56 (0.33–0.95)	0.71 (0.42–1.19)
OLN10	2.50 (1.49–4.17)	—	1.29 (0.74–2.26)	1.40 (0.70–2.81)	1.77 (0.86–3.63)
OLN5	1.96 (1.41–2.70)	0.78 (0.44–1.35)	—	1.08 (0.57–2.04)	1.37 (0.69–2.71)
OLN2.5	1.79 (1.05–3.03)	0.71 (0.36–1.43)	0.93 (0.49–1.75)	—	1.27 (0.58–2.81)
Other	1.41 (0.84–2.38)	0.57 (0.28–1.16)	0.73 (0.37–1.45)	0.79 (0.36–1.72)	—
8.3 Long-delayed					
	Placebo	OLN5			
Placebo	—	0.43 (0.29–0.65)			
OLN5	2.33 (1.54–3.45)	—			
8.4 Overall phase					
	Placebo	OLN10	OLN5	OLN2.5	Other
Placebo	—	0.40 (0.27–0.60)	0.51 (0.38–0.69)	0.58 (0.38–0.89)	0.72 (0.48–1.08)
OLN10	2.50 (1.67–3.70)	—	1.27 (0.82–1.96)	1.45 (0.88–2.39)	1.79 (1.01–3.17)
OLN5	1.96 (1.45–2.63)	0.79 (0.51–1.22)	—	1.14 (0.69–1.87)	1.41 (0.81–2.45)
OLN2.5	1.72 (1.12–2.63)	0.69 (0.42–1.14)	0.88 (0.53–1.45)	—	1.24 (0.67–2.29)
Other	1.39 (0.93–2.08)	0.56 (0.32–0.99)	0.71 (0.41–1.24)	0.81 (0.44–1.49)	—

## Appendix 9

See Table 9

**Table 9:** No nausea (odds ratio and 95% confidence intervals), subgroup analysis of highly emetogenic chemotherapy patients. Odds ratios > 1 favor the row-defining treatment

9.1 Acute phase					
	Placebo	OLN10	OLN5	OLN2.5	Other
Placebo	—	0.44 (0.22–0.88)	0.52 (0.30–0.88)	0.79 (0.36–1.75)	0.67 (0.33–1.37)
OLN10	2.27 (1.14–4.55)	—	1.18 (0.57–2.44)	1.79 (0.71–4.55)	1.52 (0.67–3.45)
OLN5	1.92 (1.14–3.33)	0.85 (0.41–1.75)	—	1.52 (0.62–3.70)	1.29 (0.57–2.94)
OLN2.5	1.27 (0.57–2.78)	0.56 (0.22–1.41)	0.66 (0.27–1.61)	—	0.85 (0.31–2.38)
Other	1.49 (0.73–3.03)	0.66 (0.29–1.49)	0.77 (0.34–1.75)	1.18 (0.42–3.23)	—
9.2 Delayed phase					
	Placebo	OLN10	OLN5	OLN2.5	Other
Placebo	—	0.39 (0.20–0.74)	0.43 (0.25–0.74)	0.70 (0.33–1.49)	0.59 (0.30–1.16)
OLN10	2.56 (1.35–5.00)	—	1.10 (0.56–2.17)	1.79 (0.76–4.17)	1.52 (0.71–3.23)
OLN5	2.33 (1.35–4.00)	0.91 (0.46–1.79)	—	1.61 (0.69–3.70)	1.37 (0.64–2.94)
OLN2.5	1.43 (0.67–3.03)	0.56 (0.24–1.31)	0.62 (0.27–1.45)	—	0.85 (0.31–2.27)
Other	1.69 (0.86–3.33)	0.66 (0.31–1.41)	0.73 (0.34–1.56)	1.18 (0.44–3.23)	—
9.3 Long-delayed phase					
	Placebo	OLN5			
Placebo	—	0.43 (0.29–0.65)			
OLN5	2.33 (1.54–3.45)	—			
9.4 Overall phase					
	Placebo	OLN10	OLN5	OLN2.5	Other
Placebo	—	0.40 (0.25–0.65)	0.45 (0.28–0.72)	0.77 (0.37–1.61)	0.61 (0.32–1.16)
OLN10	2.50 (1.54–4.00)	—	1.12 (0.67–1.89)	1.92 (0.83–4.35)	1.52 (0.76–3.03)
OLN5	2.22 (1.39–3.57)	0.89 (0.53–1.49)	—	1.72 (0.75–3.92)	1.37 (0.68–2.78)
OLN2.5	1.30 (0.62–2.70)	0.52 (0.23–1.20)	0.58 (0.26–1.33)	—	0.80 (0.30–2.13)
Other	1.64 (0.86–3.13)	0.66 (0.33–1.31)	0.73 (0.36–1.47)	1.25 (0.47–3.33)	—

## Appendix 10

See Table 10

**Table 10:** No nausea (odds ratio and 95% confidence intervals), subgroup analysis of moderately emetogenic chemotherapy patients. Odds ratios > 1 favor the row-defining treatment

10.1 Acute phase			
	Placebo	OLN10	OLN5
Placebo	—	0.58 (0.27–1.25)	0.68 (0.48–0.95)
OLN10	1.72 (0.80–3.70)	—	1.18 (0.58–2.44)
OLN5	1.47 (1.05–2.08)	0.85 (0.41–1.72)	—
10.2 Delayed phase			
	Placebo	OLN10	OLN5
Placebo	—	0.46 (0.23–0.93)	0.68 (0.49–0.93)
OLN10	2.17 (1.08–4.35)	—	1.47 (0.76–2.78)
OLN5	1.47 (1.08–2.04)	0.68 (0.36–1.31)	—
10.3 Delayed phase			
	Placebo	OLN5	
Placebo	—	0.43 (0.29–0.65)	
OLN5	2.33 (1.54–3.45)	—	
10.4 Overall phase			
	Placebo	OLN10	Oln5
Placebo	—	0.58 (0.28–1.19)	0.68 (0.50–0.93)
OLN10	1.72 (0.84–3.57)	—	1.47 (0.76–2.86)
OLN5	1.47 (1.08–2.00)	0.68 (0.35–1.31)	—

## Appendix 11

See Table 11

**Table 11:** No vomiting (odds ratio and 95% confidence intervals). Odds ratios > 1 favor the row-defining treatment

11.1 Acute phase					
	Placebo	OLN10	OLN5	OLN2.5	Other
Placebo	—	0.40 (0.24–0.67)	0.55 (0.40–0.76)	0.61 (0.36–1.03)	0.74 (0.44–1.24)
OLN10	2.50 (1.49–4.17)	—	1.38 (0.80–2.39)	1.53 (0.78–3.00)	1.84 (0.90–3.78)
OLN5	1.82 (1.32–2.50)	0.72 (0.42–1.25)	—	1.11 (0.59–2.09)	1.33 (0.68–2.58)
OLN2.5	1.64 (0.97–2.78)	0.65 (0.33–1.28)	0.90 (0.48–1.70)	—	1.20 (0.55–2.63)
Other	1.35 (0.81–2.27)	0.54 (0.26–1.11)	0.75 (0.39–1.47)	0.83 (0.38–1.82)	—
11.2 Delayed phase					
	Placebo	OLN10	OLN5	OLN2.5	Other
Placebo	—	0.40 (0.24–0.66)	0.51 (0.37–0.69)	0.57 (0.34–0.95)	0.71 (0.43–1.18)
OLN10	2.50 (1.52–4.17)	—	1.28 (0.74–2.22)	1.43 (0.73–2.80)	1.79 (0.89–3.61)
OLN5	1.96 (1.45–2.70)	0.78 (0.45–1.35)	—	1.12 (0.60–2.08)	1.40 (0.72–2.72)
OLN2.5	1.75 (1.05–2.94)	0.70 (0.36–1.37)	0.89 (0.48–1.67)	—	1.25 (0.58–2.71)
Other	1.41 (0.85–2.33)	0.56 (0.28–1.12)	0.71 (0.37–1.39)	0.80 (0.37–1.73)	—
11.3 Long-delayed					
	Placebo	OLN5			
Placebo	—	0.46 (0.30–0.70)			
OLN5	2.17 (1.43–3.33)	—			
11.4 Overall phase					
	Placebo	OLN10	OLN5	OLN2.5	Other
Placebo	—	0.40 (0.27–0.59)	0.52 (0.39–0.69)	0.59 (0.39–0.90)	0.73 (0.49–1.09)
OLN10	2.50 (1.69–3.70)	—	1.29 (0.84–1.99)	1.47 (0.89–2.43)	1.81 (1.03–3.19)
OLN5	1.92 (1.45–2.56)	0.78 (0.50–1.19)	—	1.14 (0.69–1.88)	1.40 (0.81–2.43)
OLN2.5	1.69 (1.11–2.56)	0.68 (0.41–1.12)	0.88 (0.53–1.45)	—	1.23 (0.67–2.27)
Other	1.37 (0.92–2.04)	0.55 (0.31–0.97)	0.71 (0.41–1.23)	0.81 (0.44–1.49)	—

## Appendix 12

See Table 12

**Table 12:** No vomiting (odds ratio and 95% confidence intervals), subgroup analysis of highly emetogenic chemotherapy patients. Odds ratios > 1 favor the row-defining treatment. **12.1** Acute Phase **12.2** Delayed Phase **12.3** Long-Delayed **12.4** Overall Phase

12.1 Acute phase					
	Placebo	OLN10	OLN5	OLN2.5	Other
Placebo	—	0.39 (0.21–0.74)	0.48 (0.29–0.79)	0.74 (0.34–1.61)	0.61 (0.31–1.22)
OLN10	2.56 (1.35–4.76)	—	1.23 (0.67–2.27)	1.92 (0.83–4.35)	1.56 (0.76–3.23)
OLN5	2.08 (1.27–3.45)	0.81 (0.44–1.49)	—	1.56 (0.68–3.57)	1.27 (0.61–2.63)
OLN2.5	1.35 (0.62–2.94)	0.52 (0.23–1.20)	0.64 (0.28–1.47)	—	0.82 (0.31–2.17)
Other	1.64 (0.82–3.23)	0.64 (0.31–1.31)	0.79 (0.38–1.64)	1.22 (0.46–3.23)	—
12.2 Delayed phase					
	Placebo	OLN10	OLN5	OLN2.5	Other
Placebo	—	0.39 (0.20–0.71)	0.45 (0.27–0.74)	0.71 (0.34–1.49)	0.59 (0.30–1.16)
OLN10	2.56 (1.41–5.00)	—	1.16 (0.63–2.13)	1.82 (0.80–4.17)	1.52 (0.74–3.13)
OLN5	2.22 (1.35–3.70)	0.86 (0.47–1.59)	—	1.57 (0.69–3.57)	1.31 (0.64–2.70)
OLN2.5	1.41 (0.67–2.94)	0.55 (0.24–1.25)	0.64 (0.28–1.45)	—	0.83 (0.31–2.22)
Other	1.69 (0.86–3.33)	0.66 (0.32–1.35)	0.76 (0.37–1.56)	1.20 (0.45–3.23)	—
12.3 Long-delayed					
	Placebo	OLN5			
Placebo	—	0.46 (0.30–0.70)			
OLN5	2.17 (1.43–3.33)	—			
12.4 Overall phase					
	Placebo	OLN10	OLN5	OLN2.5	Other
Placebo	—	0.40 (0.24–0.64)	0.46 (0.29–0.72)	0.77 (0.37–1.61)	0.61 (0.32–1.16)
OLN10	2.50 (1.56–4.17)	—	1.15 (0.69–1.92)	1.92 (0.83–4.35)	1.52 (0.76–3.03)
OLN5	2.17 (1.39–3.45)	0.87 (0.52–1.45)	—	1.67 (0.73–3.85)	1.31 (0.65–2.63)
OLN2.5	1.30 (0.62–2.70)	0.52 (0.23–1.20)	0.60 (0.26–1.37)	—	0.79 (0.30–2.08)
Other	1.64 (0.86–3.13)	0.66 (0.33–1.31)	0.76 (0.38–1.54)	1.27 (0.48–3.33)	—

## Appendix 13

See Table 13

**Table 13:** No vomiting (odds ratio and 95% confidence intervals), subgroup analysis of moderately emetogenic chemotherapy patients. Odds ratios > 1 favor the row-defining treatment

13.1 Acute phase			
	Placebo	OLN10	OLN5
Placebo	—	0.58 (0.27–1.25)	0.68 (0.48–0.95)
OLN10	1.72 (0.80–3.70)	—	1.18 (0.58–2.44)
OLN5	1.47 (1.05–2.08)	0.85 (0.41–1.72)	—
13.2 Delayed phase			
	Placebo	OLN10	OLN5
Placebo	—	0.46 (0.23–0.93)	0.68 (0.49–0.93)
OLN10	2.17 (1.08–4.35)	—	1.47 (0.76–2.78)
OLN5	1.47 (1.08–2.04)	0.68 (0.36–1.31)	—
13.3 Long-delayed			
	Placebo	OLN5	
Placebo	—	0.46 (0.30–0.70)	
OLN5	2.17 (1.43–3.33)	—	
13.4 Overall phase			
	Placebo	OLN10	OLN5
Placebo	—	0.58 (0.28–1.19)	0.68 (0.50–0.93)
OLN10	1.72 (0.84–3.57)	—	1.47 (0.76–2.86)
OLN5	1.47 (1.08–2.00)	0.68 (0.35–1.31)	—

## Appendix 14

See Table 14

**Table 14:** Safety endpoints (odds ratio and 95% confidence intervals). Odds ratios > 1 favor the row-defining treatment

14.1 Sedation					
	Placebo	OLN10	OLN5	OLN2.5	Other
Placebo	—	0.61 (0.35–1.07)	0.42 (0.24–0.74)	0.55 (0.28–1.10)	0.78 (0.39–1.56)
OLN10	1.64 (0.94–2.86)	—	0.69 (0.36–1.33)	0.91 (0.41–2.02)	1.28 (0.53–3.09)
OLN5	2.38 (1.35–4.17)	1.45 (0.75–2.78)	—	1.31 (0.58–2.98)	1.83 (0.75–4.48)
OLN2.5	1.82 (0.91–3.57)	1.10 (0.50–2.43)	0.76 (0.34–1.73)	—	1.40 (0.52–3.77)
Other	1.28 (0.64–2.56)	0.78 (0.32–1.89)	0.55 (0.22–1.34)	0.71 (0.27–1.93)	—
14.2 Diarrhea					
	Placebo	OLN10	OLN5	Other	
Placebo	—	0.70 (0.23–2.10)	0.41 (0.19–0.90)	1.50 (0.23–9.42)	
OLN10	1.43 (0.48–4.35)	—	0.59 (0.17–2.01)	2.14 (0.28–16.3)	
OLN5	2.44 (1.11–5.26)	1.69 (0.50–5.88)	—	2.21 (0.29–16.8)	
Other	0.67 (0.11–4.35)	0.47 (0.06–3.57)	0.45 (0.06–3.45)	—	
14.3 Headache					
	Placebo	OLN10	OLN5	Other	
Placebo	—	1.81 (0.57–5.76)	1.13 (0.45–2.83)	1.54 (0.41–5.80)	
OLN10	0.55 (0.17–1.75)	—	0.62 (0.18–2.11)	0.85 (0.18–3.94)	
OLN5	0.88 (0.35–2.22)	1.62 (0.47–5.56)	—	1.36 (0.31–5.96)	
Other	0.65 (0.17–2.44)	1.18 (0.25–5.56)	0.74 (0.17–3.23)	—	
14.4 Constipation					
	Placebo	OLN10	OLN5	Other	
Placebo	—	3.82 (0.71–20.41)	1.36 (0.86–2.17)	1.21 (0.69–2.13)	
OLN10	0.26 (0.05–1.41)	—	0.36 (0.06–2.04)	0.32 (0.05–1.88)	
OLN5	0.74 (0.46–1.16)	2.78 (0.49–15.9)	—	0.89 (0.50–1.58)	
Other	0.83 (0.47–1.45)	3.13 (0.53–18.2)	1.12 (0.63–2.00)	—	
14.5 Appetite loss					
	Placebo	OLN10	OLN5	OLN2.5	Other
Placebo	—	0.75 (0.40–1.41)	0.57 (0.27–1.22)	0.69 (0.25–1.88)	0.82 (0.29–2.34)
OLN10	1.34 (0.71–2.51)	—	0.76 (0.29–1.99)	0.92 (0.28–3.04)	1.10 (0.31–3.89)
OLN5	1.76 (0.82–3.76)	1.31 (0.50–3.45)	—	1.21 (0.32–4.61)	1.45 (0.36–5.82)
OLN2.5	1.45 (0.53–4.00)	1.09 (0.33–3.57)	0.83 (0.22–3.13)	—	1.20 (0.20–7.08)
Other	1.22 (0.43–3.45)	0.91 (0.26–3.23)	0.69 (0.17–2.78)	0.83 (0.14–5.00)	—
14.6 Hiccups					
	Placebo	OLN10	OLN5		
Placebo	—	0.89 (0.29–2.74)	1.44 (0.58–3.55)		
OLN10	1.12 (0.36–3.45)	—	1.62 (0.42–6.24)		
OLN5	0.69 (0.28–1.72)	0.62 (0.16–2.38)	—		
